# Cerebellar acceleration of learning in an evidence-accumulation task

**DOI:** 10.1016/j.celrep.2026.117262

**Published:** 2026-04-16

**Authors:** Marlies Oostland, Mikhail Kislin, Yuhang Chen, Tiffany Chen, Sarah Jo Venditto, Ben Deverett, Samuel S.-H. Wang

**Affiliations:** 1 Neuroscience Institute, Princeton University, Princeton, NJ, USA; 2 Swammerdam Institute for Life Sciences, University of Amsterdam, Amsterdam, the Netherlands; 3 Department of Neurological Surgery, University of California, San Francisco, San Francisco, CA, USA; 4 Department of Anesthesiology, Stanford University Medical Center, Stanford, CA, USA; 5 Present address: Albert Einstein College of Medicine, 1410 Pelham Parkway S, Kennedy 915, Bronx, NY, USA; 6 These authors contributed equally; 7 Lead contact

## Abstract

Cerebellar processing contributes to sensory salience, cognition, and behavioral flexibility. Here, we report that learning on a sensory evidence-accumulation task in mice is accelerated by cue-locked optogenetic stimulation of Purkinje cells but not by continuous optogenetic interference. Latent-state analysis revealed that accelerated learning was associated with enhanced focus on current over past trials. A cerebellum-specific transgenic autism model with disrupted Purkinje cell function also unexpectedly showed accelerated learning as well as enhanced reactivity to touch and auditory cues. Transgenic mice and wild-type mice receiving cue-locked stimulation showed prolonged sensory responses in Purkinje cell complex spikes and anterior cingulate cortex, and a subset of Purkinje cells in crus I showed on-task enhanced response to stimuli in wild-type mice. Sensory salience and task state may be different facets of a complex-spike-based mechanism for regulating brain-wide learning mechanisms. These findings potentially link cerebellum-dependent sensory salience with a global weak coherence account of autism.

## In brief

Sensory cue-locked optogenetic perturbation of Purkinje cells leads to faster learning of a working memory task mediated by higher focus on current trials. The effects are emulated by a cerebellum-based mouse autism model, and both perturbations drive enhanced neocortical activity. Results are consistent with a weak coherence model for autism.

## Graphical Abstract

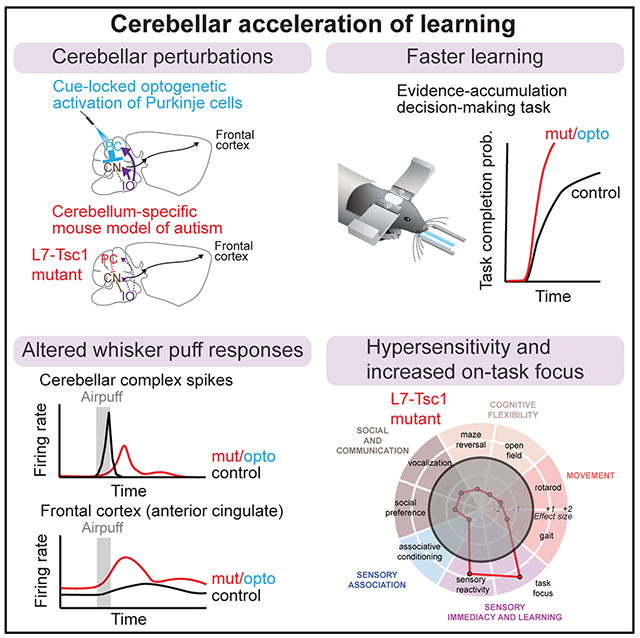

## INTRODUCTION

The cerebellum’s roles extend beyond movement to include sensory processing, cognition, learning, and memory.^1–3^ Recent neuroimaging, clinical, and animal research provides evidence for a cerebellar role in social cognition and adaptive prediction,^4–6^ and in mice, cerebellar disruption can lead to deficits in attention, behavioral flexibility, and social interaction.^7^ Functional effects can be long lasting, since early-life cerebellar injury in humans leads to autism spectrum disorder (ASD) and other nonmotor disabilities.^8–12^

A shared feature of sensory and cognitive processing is the role of event salience. In classical computational models of learning,^13^ the salience of sensory events is assumed to be constant, leading to an unchanging effect on the rate and amount of learning. In fact, however, the salience of events is actively regulated. A key component of salience is the degree of expectedness of a stimulus. The cerebellum encodes unexpected events via complex spikes to drive simple associative learning.^14^ Complex spikes can drive transient changes in firing, including rebound, in deep nuclear neurons,^15,16^ which project to midbrain including thalamocortical systems. Thus, complex spikes represent a means for representing sensory salience and conveying it to the rest of the brain.

Here, we present evidence for enhanced task learning arising from cerebellum-specific functional perturbation. To examine alterations in task performance that emerge from abnormal cerebellar circuits, we examined acute optogenetic perturbations of cerebellar activity and found accelerated learning on an evidence-accumulation task. We also found, surprisingly, that *L7-Tsc1* mutants, representing a mouse model in which *tuberous sclerosis complex 1* is deleted specifically in cerebellar Purkinje cells and leads to decreased firing output,^17,18^ also showed accelerated learning. Further investigations revealed a shared mechanism in which complex-spike output putatively regulates sensory reactivity and salience at brain-wide scale to support task persistence and learning.

## RESULTS

**Figure 1.**
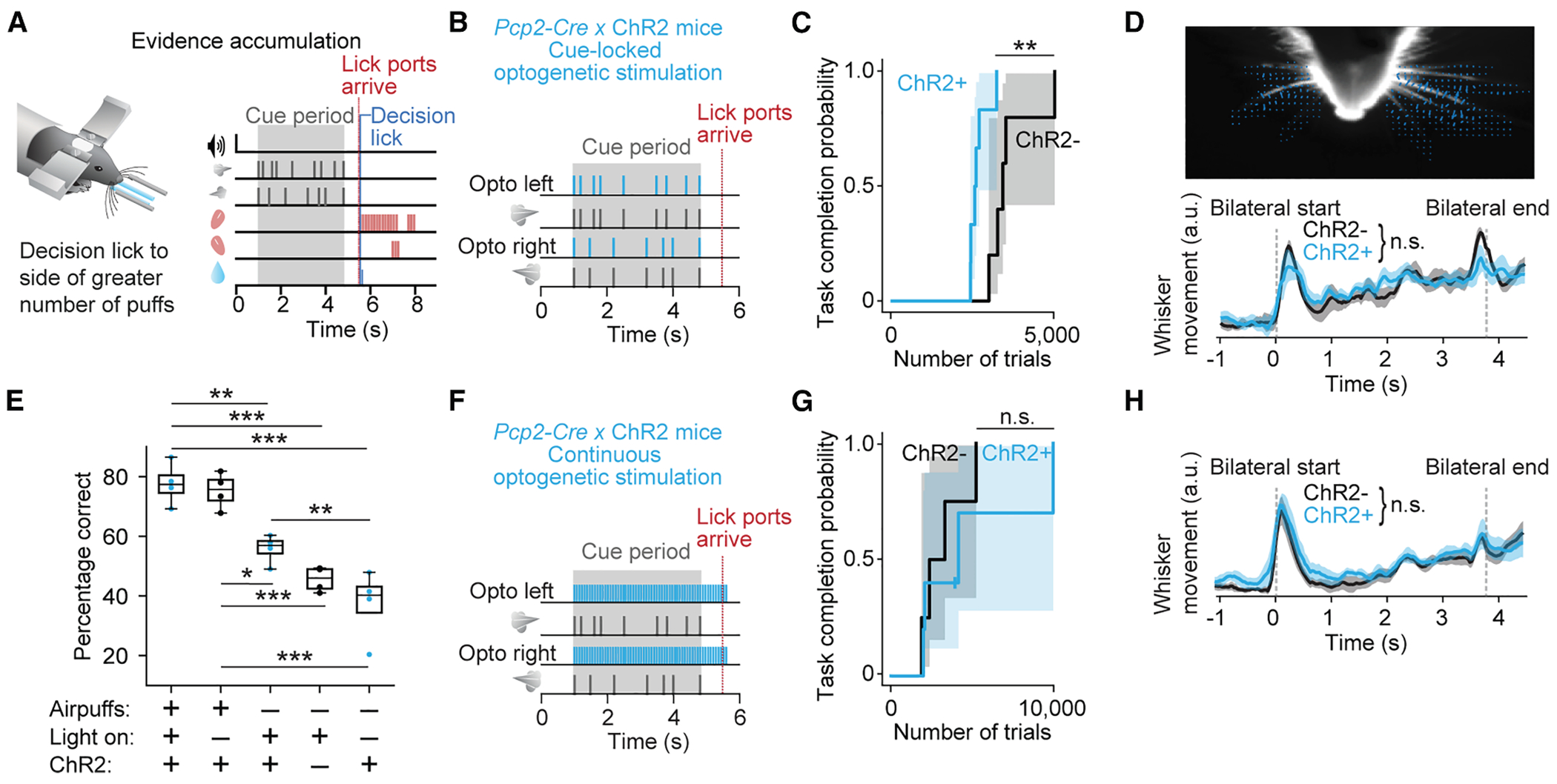
Optogenetic manipulation of cerebellar crus I affects learning rate, but not sensory responses, on an evidence-accumulation task (A) Schematic of the evidence-accumulation task. Mice receive sensory airpuffs on the left and right whiskers and receive a reward for correctly licking in the direction of more puffs. (B) The evidence-accumulation task with cue-locked optogenetic stimulation. (C) Kaplan-Meier estimator of training completion for six *Pcp2-Cre* × ChR2 mice with cue-locked bilateral optogenetic activation of crus I, compared with five wild-type littermates (log-rank test). (D) Detection of whisker movement during the evidence-accumulation task with cue-locked optogenetic activation. Blue arrows indicate detection of whisker movement measured using a region-of-interest optical flow analysis. (E) Performance in the evidence-accumulation task in four trained *Pcp2-Cre* × ChR2 mice for trials at level 7 under various experimental conditions, from left to right: airpuffs combined with cue-locked optogenetic activation of Purkinje cells in crus I, airpuffs only, optogenetic activation only, and negative controls lacking ChR2 or optogenetic stimuli. Due to anti-biasing parameters, chance-level performance for each condition was always less than 50%. Boxes show median/ interquartile range, and whiskers extend to the farthest data point that lies within 1.5 times the interquartile range from the end of the box. Comparisons were made using a Conover post hoc test. (F) The evidence-accumulation task with continuous bilateral optogenetic stimulation. (G) Kaplan-Meier estimator of training completion probability for 5 *Pcp2-Cre* × ChR2 mice with continuous bilateral optogenetic activation of crus I throughout the evidence-accumulation task compared to four wild-type littermates. (H) Detection of whisker movement during the evidence-accumulation task with continuous bilateral optogenetic activation. In (C), (D), (G), and (H), shaded areas include 95% confidence intervals (CIs). *p ≤ 0.05, **p ≤ 0.01, ***p ≤ 0.001., n.s., not significant.

To examine cerebellum-dependent sensory processing, we used a complex task in which mice learn to integrate sensory evidence in working memory using an established evidence-accumulation decision-making paradigm^19,20^ (Figure 1A). Post-learning performance of this task depends on cerebellar crus I,^19,21^ a region that is also necessary for other nonmotor functions^7^ including visuomotor reinforcement learning.^22^ During the task, mice receive sensory airpuffs on the left and right whiskers and receive a reward for correctly licking in the direction of more puffs (Video S1). Mice progress through increasingly complex levels of task shaping: puffs come to be presented on both left and right, then the difference between the number of puffs on each side becomes smaller, then an increasing temporal delay separates the end of sensory information from the decision lick (Figure S1 and Table S1). As expected, the fraction of correct trials dips when animals transition to the next level of the task, and not all animals reach the final stage, consistent with the task becoming increasingly difficult with advancing levels.

### Cue-locked optogenetic stimulation boosts learning rate

To determine whether perturbation of the cerebellum could enhance learning, we manipulated neural activity in wild-type mice by expressing the optogenetic probe channelrhodopsin-2 (ChR2) in Purkinje cells (Figure 1B). We optogenetically reinforced each cue, starting after mice had passed out of the early stage of training, by pairing each sensory stimulus with a cue-locked ipsilateral optogenetic light flash applied over crus I, a stimulus that evokes a complex spike directly in targeted Purkinje cells.^23^

As a means of monitoring training progress, we graphed the time course of learning as a Kaplan-Meier plot (Figure 1C), a method for displaying and analyzing event-occurrence data that allowed us to use tick marks to display mice that failed to complete training by a particular point in time (“censoring” events^24^; see tick marks on data curves in Figures 5C and 5G; see also Figure S3A). Cue-locked ipsilateral flashes led to faster learning than in strain-matched controls not expressing ChR2 (Figure 1C; median 2,512 trials in six mice, compared with 3,311 trials in five wild-type littermates; *χ*^2^_(10)_ = 8.18, *p* = 0.0042, log-rank test).

Optogenetically driven acceleration of learning might arise from enhancement of direct sensory responsiveness or, alternatively, add an additional signal that shapes learning. In trained mice, the shape of the psychometric curves in each state was similar with or without cue-locked optogenetic stimulation (Figures S2A and S2B). In a sensory reactivity test, cue-locked optogenetic stimulation did not increase the number of blinks produced to higher-intensity airpuffs (within-mouse comparison; 25-psi puffs of 8- to 45-ms duration, *F*_(2,48)_ = 0.23, *p* = 0.80 for cue-locked flashes vs. no flashes, linear mixed-effects [LME] model, five mice). The amount of per-puff whisker movement in the evidence-accumulation task was also not affected (Figure 1D, rise of total frame-on-frame changed pixels during the cue period: *p* = 0.27; decay: *p* = 0.53; two-tailed Welch’s *t* test, *n* = 5 ChR^−^ mice and *n* = 6 ChR^+^ mice), indicating that the accelerated learning that occurs with cue-locked optogenetic stimulation drives mechanisms that are in some way separate from immediate sensorimotor processing.

We found that after training under cue-locked optogenetic enhancement to the highest level of task, mice were able to do the task both with airpuffs + optogenetic stimuli (77.6% correct) and with airpuffs only (75.3% correct; Figure 1E). We were interested to observe that they could also perform above chance with optogenetic stimuli only (opto-only, 55.8%). Opto-only performance was, in turn, better than mice not expressing ChR2 (45.6% correct, *p* = 0.046, Conover post hoc test) or better than mice receiving neither airpuffs nor optogenetic stimuli (37.2% correct, *p* = 0.0044; note that anti-biasing can bring chance performance below 50%). Together, these observations show that in well-trained mice, cerebellar activity alone (as indicated by the opto-only trials) is sufficient to play an autonomous role in evidence accumulation but that, under such conditions, optogenetic enhancement of cerebellar activity does not enhance the role of sensory evidence.

Continuous optogenetic stimulation of Purkinje cells in crus I in trained mice during the cue period and delay period including the first lick (Figure 1F) is known to impair performance through the forgetting of accumulated sensory evidence.^21^ Unlike cue-locked stimulation, this perturbation had no detectable effect on the learning rate (Figure 1G) (*Pcp2-Cre* × ChR2 animals: median 4,210 trials, *n* = 5; wild-type littermates: median 2,534 trials, *n* = 4; *χ*^2^_(8)_ = 0.31, *p* = 0.33, log-rank test) or on whisker movements before, during, or after the cue period (Figure 1H) (rise: *p* = 0.61; decay: *p* = 0.33; two-tailed Welch’s *t* test, *n* = 4 ChR^−^ mice and *n* = 5 ChR^+^ mice). Psychometric curves in trained mice were also not affected by continuous optogenetic activation (Figures S2C and S2D). Thus, unlike cue-locked activation, generalized continuous activation of crus I does not accelerate learning.

### Latent behavioral-state analysis of task learning reveals bouts of high performance and on-task focus

**Figure 2.**
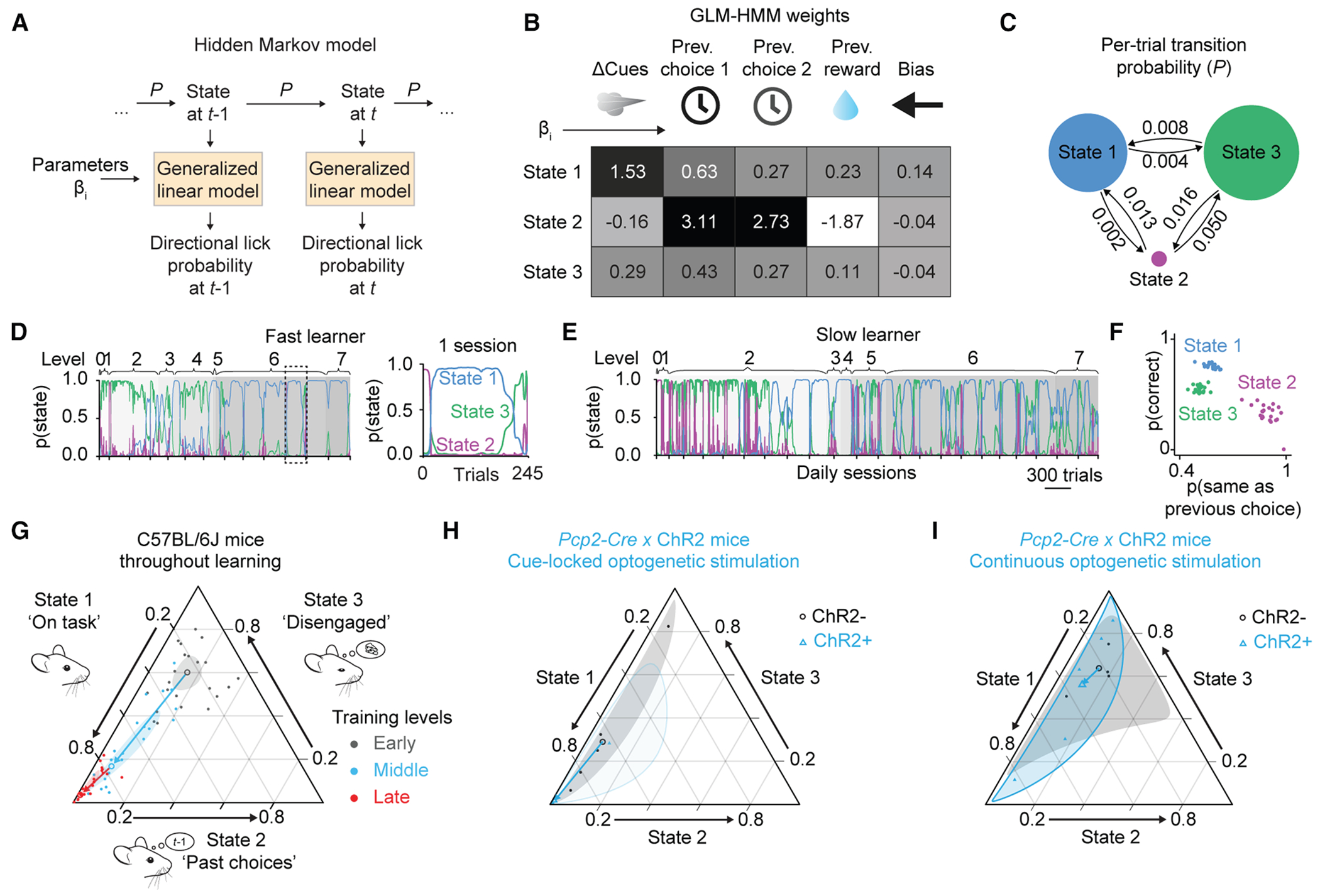
Latent behavioral-state analysis of task learning reveals periods of high performance and on-task focus (A) Schematic of the generalized linear model-hidden Markov model (GLM-HMM). *P* is state-transition probability. (B) Inferred GLM-HMM weights from the training dataset. (C) Per-trial transition rate between the three states averaged over all mice. The size of the circles indicates state occupancy across all trials of the mice in the training dataset. (D) Posterior state probabilities for all trials in all sessions (left) and one example session (right) from a fast learner. Area bound by the dashed area in the left panel indicates the session in the right panel. The left panel is on the same time scale as in (E). (E) Posterior state probabilities for all trials in all sessions from a slow learner. Scale bar, 300 trials. (F) Probability of a correct choice against the probability that the choice in the current trial was the same as the choice in the previous trial. Each data point represents the average across all trials for one mouse. (G–I) Ternary plots for the composition of latent states with 95% confidence bands (shaded regions) around the compositional mean (open symbols) for C57BL/6J mice across early, middle, and late training levels (G), *Pcp2-Cre* × ChR2 mice with cue-locked optogenetic activation of crus I (H), and *Pcp2-Cre* × ChR2 mice with continuous bilateral optogenetic activation of crus I (I). Each data point represents one mouse. The 95% confidence regions are based on an assumption of normality for compositions. Arrows indicate the shift between averages.

To explore alterations in behavior associated with accelerated learning, we performed computational latent-state analysis of the learning process throughout training (Figure 2). Latent-state analysis identifies shifts in behavioral response patterns occurring between groups of trials that reflect changes in internal state over time.^25–27^ We fitted trial-by-trial outcomes using generalized linear modeling of a hidden Markov model (GLM-HMM;Figure 2A). The fit was done using previously developed methods of machine learning to define a GLM-HMM.^28^ In our GLM-HMM, within a training session a mouse is assumed to pass among a user-specified number of states with a fixed trial-to-trial probability. In each of these states, the correct/incorrect performance probability is a function of task parameters (here current cues, previous trials, and bias). To attain out-of-sample independence for the current behavioral dataset, we used a training set of nearly 98,000 trials from 22 wild-type mice from a previous study^19,21^ to fit a single set of parameters, including state-to-state transition probabilities and a softmax function for converting parameters to performance probability. The likelihood function of the GLM-HMM to capture mouse behavior reached a plateau for three or more states, and we chose a three-state model for further analysis.

The three states of mouse behavior during the task differed in their dependence on task parameters (Figure 2B). Mice in the on-task state 1 made the most correct decisions, relying heavily on the left-right difference in sensory cues, and less on the animal’s choice in the previous trial. Early in training, wild-type mice tended to spend time in state 2, a past-trial-driven state in which they relied more on past rather than present information, thus reducing their decision accuracy, with responses strongly dependent on the choices made in the previous two trials; and in state 3, an inattentive state in which they made choices but were only weakly sensitive to any task-specific events. On a moment-to-moment basis, wild-type mice made transitions from state to state (Figure 2C) on the timescale of dozens or hundreds of trials (Figures 2D and 2E). Across sessions as training progressed, animals gradually shifted away from state 2 or state 3 occupancy, eventually reaching consistent state 1 occupancy (Figures 2D and 2E). Within sessions, transitions away from state 1 occurred largely at the end of the session, when animals typically switched from the on-task state 1 to the disengaged state 3 (see example in Figure 2D). Each of these states had a dependency on past trials (Figure 2F), and mice spent progressively more time in state 1 as training progressed (Figure 2G). This shift in state occupancy occurred across all animals (compositional analysis of variance on the log ratio state 2/state 1, significant effect for training level, *F*_(2)_ = 53.9, *p* < 0.001; for detailed comparisons, see Figure 2G).

We then fitted data from the experimental animals to this model. We found that cue-locked optogenetic activation had specific effects on the hidden behavioral state of mice performing the task. By late training, four out of five optogenetically reinforced mice spent more than 90% of the trials in state 1 and fewer than 5% of the trials in state 2 (Figure 2H). Analysis of variance on the log ratio state 2/state 1 yielded a significant difference for both group (*F*_(1)_ = 12.3, *p* = 0.003) and training level (*F*_(1)_ = 16.4, *p* < 0.001).

To measure how differential occupancy of task states evolves over training, we made use of our division of the behavioral data into three stages of task shaping: early (levels 0–2), middle (levels 3–4), and late (levels 5–6) (Figure S1). Post hoc comparisons using the Tukey honest-significant-difference (HSD) test detected no difference in log ratio state 2/state 1 at middle stages of training (levels 3–4, the earliest levels at which the manipulation occurred, *p* = 0.43) and a decrease by late stages of training (levels 5–6, *p* = 0.02). The log ratio state 3/state 1 was lower in the optogenetically reinforced mice than in animals not expressing ChR2 (*F*_(1)_ = 10.3, *p* = 0.006), without a difference by level (*F*_(1)_ = 0.09, *p* = 0.77). Continuous optogenetic activation did not lead to a change in state 1 occupancy, although there was a reduction in state 2 occupancy that was visible starting at the earliest stages of perturbation (Figure 2I). In summary, cue-locked optogenetic stimulation led to a specific gradual enhancement in the occupancy of the on-task state 1, which grew as training progressed.

### In off-task states, accumulated sensory information is noisy and leaky

**Figure 3.**
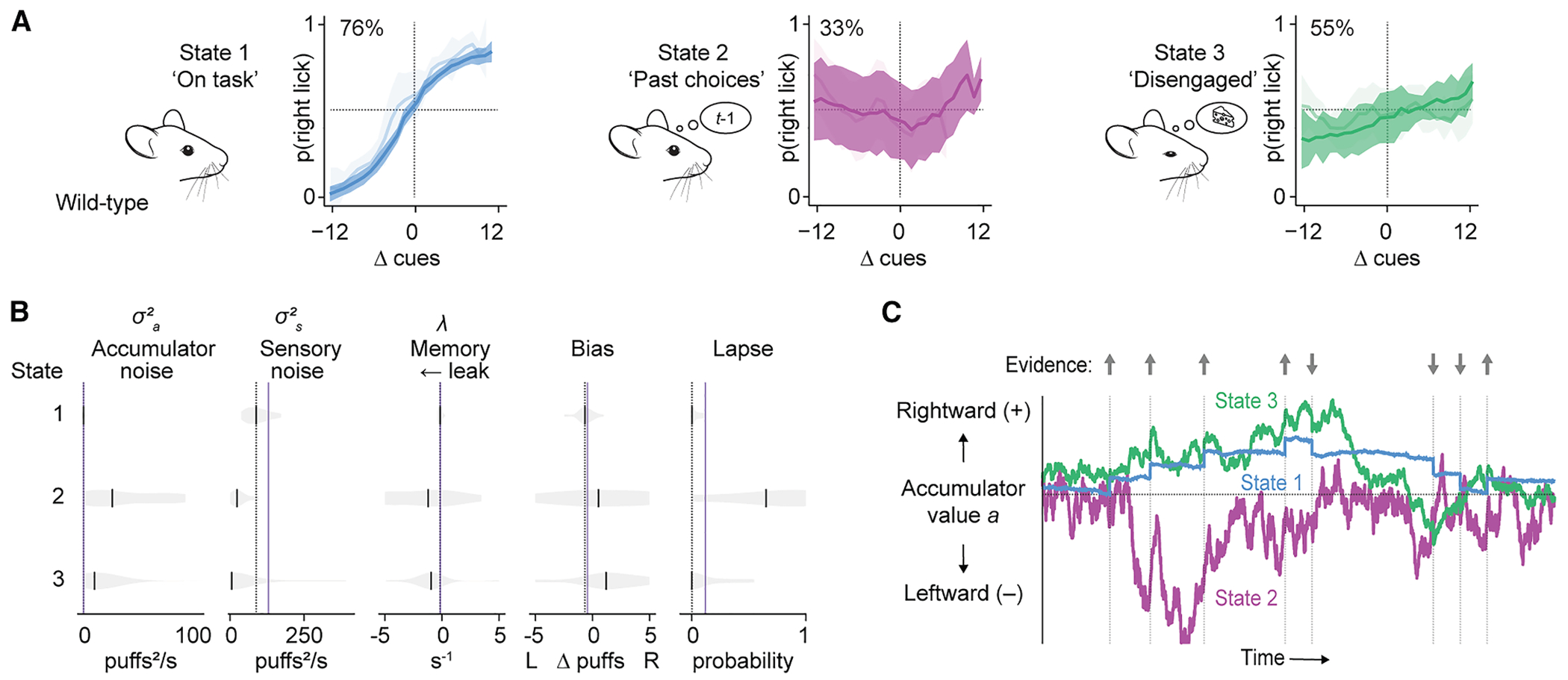
State-dependent processing and retention of sensory evidence (A) Psychometric curves averaged across all mice for the three different behavioral latent states. At the top left is the percentage correct over all trials in that state. Shaded areas represent 1 SD. Lighter curves indicate early trials, and darker curves indicate late trials. (B) Best-fit drift-diffusion model parameters for the three different behavioral latent states. Fits were computed multiple times for each condition using random subsets of the data to assess the reliability of the best-fit parameters. Black vertical ticks indicate the median best-fit parameter across fit repetitions. Gray shading represents the distribution of fit parameters across repetitions. Vertical purple lines denote best-fit values in one of our earlier datasets.^21^ (C) Simulation of the drift-diffusion model. The model’s accumulator value a is shown as it evolves over time in a single behavioral trial. Colored lines demonstrate how the trajectory of a is qualitatively altered in the three different states. Arrows and associated vertical lines indicate pulses of evidence.

Each latent state had a distinct psychometric performance curve (Figure 3A). We therefore sought to understand how the states differed in the processing and retention of sensory evidence. We fitted performance data of wild-type C57BL/6J mice within each state to a drift-diffusion model we previously used^21^ to parameterize the within-trial contributions of sensory noise, accumulator noise, and stability of information storage (Figure 3B).

We found that state 1’s drift-diffusion parameters were consistent with prior observations in high-performing mice (vertical purple lines replotted from Deverett et al.^21^). Accumulator noise was low, and the accumulated information was stored with a stable time constant of 5.9 ± 1.1 s (leak rate −0.17 ± 0.03 s^−1^, median ± SEM; Table S2), comparable to previously observed storage times. In contrast, off-task states 2 and 3 showed high accumulator noise, and accumulated information leaked away rapidly in both state 2 (time constant 0.8 ± 0.6 s, leak rate −1.20 ± 0.88 s^−1^) and state 3 (time constant 1.1 ± 0.4 s, leak rate −0.95 ± 0.34 s^−1^), indicating an emphasis on late-occurring stimuli within each trial. In state 2, the lapse rate was high (0.65 ± 0.12), consistent with the emphasis on irrelevant past trials identified using GLM-HMM analysis. These off-task parameter fits were in a range comparable with those in previous experiments in which the evidence accumulation and storage process were disrupted by optogenetic interference in crus I.^21^ Simulation of the fitted drift-diffusion parameters shows differences in the integration of evidence among the three states (Figure 3C). Taken together, these findings suggest that whether a mouse is in a state of either excessively attending to past trials (state 2) or disengaging from the task parameters (state 3), it fails to use mechanisms of evidence accumulation and storage that we have previously shown to require normal cerebellar activity.

### Task-related complex-spike responses vary systematically across Purkinje cell dendrites

**Figure 4.**
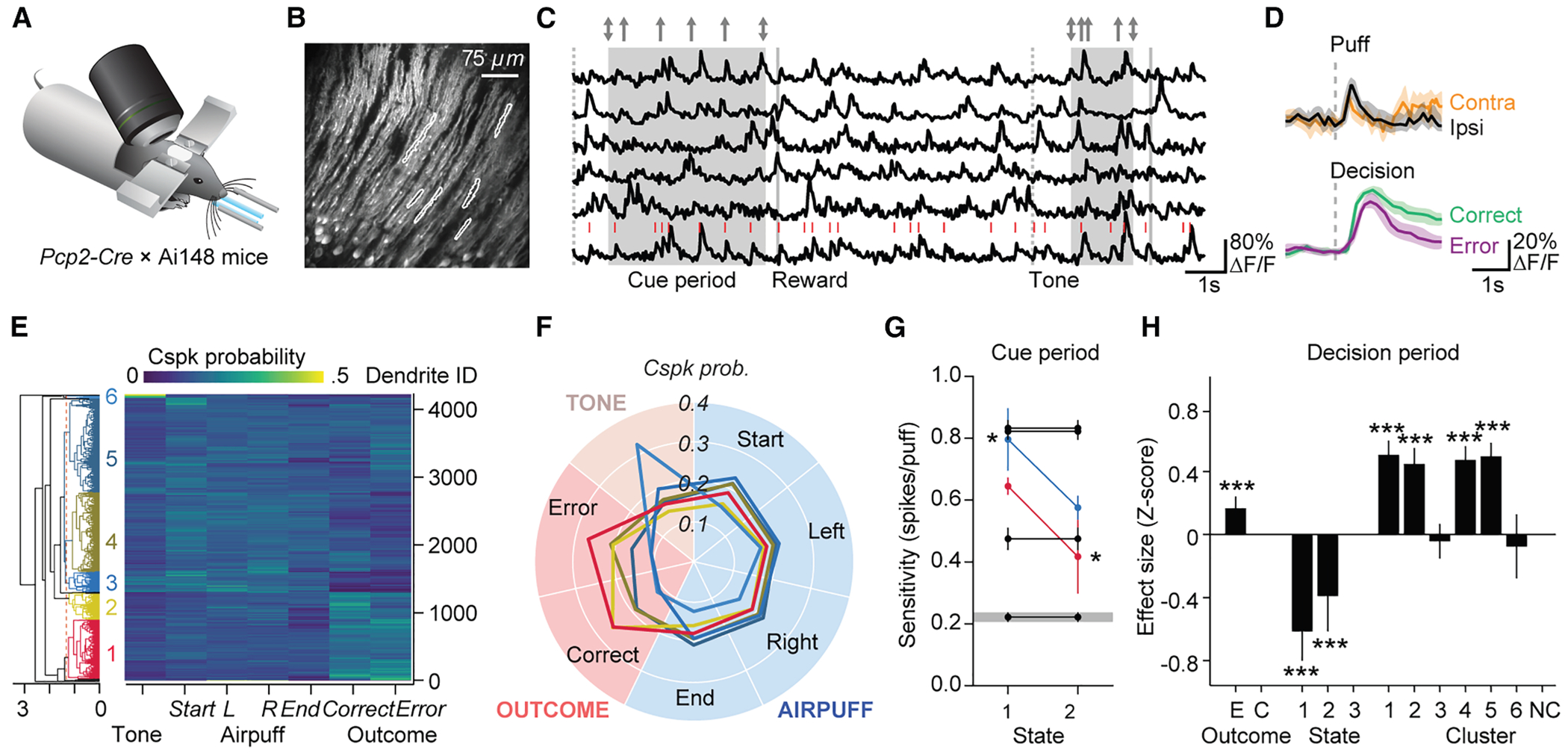
Task-related complex-spike responses vary with latent behavioral state (A) Schematic of *Pcp2-Cre* × Ai148 animal executing the evidence-accumulation task during two-photon imaging. (B) Example two-photon field of view of Purkinje cell dendrites. Scale bar represents 75 μm. (C) GCaMP6f signals extracted from dendrites indicated in (B). Dashed lines indicate tone presentation at the start of trials, shaded regions indicate cue periods, arrows at the top denote cue timing and side, and gray lines mark the timing of decision licks. Red ticks represent dendritic calcium transients extracted from the bottom trace. (D) Top: mean activity of an example dendrite aligned to the moment of individual airpuffs. Bottom: mean response of an example dendritic signal aligned to decision licks. Shading indicates 95% CI. (E) Left: dendrogram showing hierarchical clustering results for task-related complex-spike signals. Right: heatmap displaying mean response probability of each dendrite at 0–167 ms from the onset of trial events. Cspk, complex spike. (F) Mean task-event tuning for dendrites classified into each cluster in (E). Colors refer to the colors of the clusters in the left panel of (E). (G) State dependence of complex-spike response per airpuff during the cue period, as measured by the sum of significant main and higher-order effects (post hoc adjusted means) in a linear mixed model. Asterisks indicate statistically significant three-way interaction effects for state × puffs × cluster (three-way ANOVA, Satterthwaite approximation, *p ≤ 0.05). Only clusters with significant three-way interaction effects are colored. Colors refer to the colors of the clusters in the left panel of (E): red is cluster 1, and blue is cluster 3. Gray shading indicates the reference level of the linear mixed model. (H) Main effects from a linear mixed model of complex-spike activity during the post-decision period (ANOVA, Satterthwaite approximation, ***p ≤ 0.001). E, error; C, correct outcome; NC, not clustered. Values indicate differences from correct outcome, state 3, and unclustered dendrites as reference values.

Generally, complex spikes are evoked by unexpected events to shape action. In the evidence-accumulation task, task-related sensory events trigger complex-spike activity in crus I during performance in both the cue period and the decision/reward period.^19^ To test whether complex-spike responsiveness varied systematically with behavioral state, we used multiphoton *in vivo* microscopy (Figures 4A–4C) to measure GCaMP6f-based calcium transients in 4,224 Purkinje cell dendrites from 3 *Pcp2-Cre* × Ai148 mice over 13 sessions of training (see Video S1).

We assessed dendritic calcium transients in response to task-related events: auditory tone, bilateral puffs at the cue-period onset and offset, ipsi- and contralateral airpuffs, and decision lick. Of these events, decision licks (Figure 4D) and bilateral puffs at the cue-period onset (Figure 4E) showed the most prominent associated calcium transients. For each imaged dendrite, we calculated the probability of a dendritic calcium transient within a 167-ms window following these events, pooling trials within an imaging session to obtain a within-session probability. Response probabilities were then normalized to a sum of 1 across event types and hierarchically clustered. Clustering revealed six main groups (Figure 4E) comprising 98% of all dendrites, which responded most strongly to decision licks (clusters 1 and 2, 21% and 9% of dendrites, respectively), ipsilateral puffs (cluster 3, 7% of dendrites), generally to all events (clusters 4 and 5, 28% and 32% of dendrites, respectively), and auditory tone (cluster 6, 1% of dendrites) (Figure 4F). The remaining 2% of dendrites were not clustered.

### Latent behavioral state regulates the pattern of evidence representation across dendrites

To examine state-dependent responses to sensory evidence during the cue period, we analyzed 2,740 dendrites that had sufficient trials across behavioral states. We used an LME model (969,626 observations, AIC = 4.769 × 10^6^) to fit the total number of dendritic calcium transients during the cue period (putative complex spikes, *C*_cue_) to a function of total number of puffs (bilateral and unilateral puffs), categorical variables for state (reference: state 3) and cluster (reference: unclassified dendrites), and interaction effects of puffs × state × cluster, with random effects for mouse and dendrite.

The mixed-effects model revealed three significant effects. There was a strong main effect of puffs on *C*_cue_ (*F*_(1, 35.64)_ = 206.1, *p* < 10^−12^, ANOVA marginal test, Satterthwaite criterion), indicating that the number of puffs strongly influenced neural responses. We also found a highly significant main effect of cluster (*F*_(6, 247.48)_ = 237.58, *p* < 10^−12^), showing that different neural clusters had distinctive baseline activity levels. Finally, a significant puffs × cluster interaction (*F*_(6, 603.25)_ = 183.42, *p* < 10^−12^) demonstrated variation in the responsiveness of different clusters to puffs.

Although state by itself had little or no effect on per-puff sensitivity (state × puffs, *F*_(2, 10780)_ = 0.66, *p* = 0.518) or baseline activity (*F*_(2, 9913.6)_ = 2.76, *p* = 0.064), we did observe a highly significant state × cluster interaction (*F*_(12, 8816.7)_ = 7.12, *p* < 10^−12^) as well as a three-way interaction between state, puffs, and cluster (*F*_(12, 9618.9)_ = 9.34, *p* < 10^−12^). Thus, the distributions of both baseline and per-puff complex-spike activity were regulated across clusters in a behavioral-state-dependent manner.

In particular, two interactions with behavioral state affected cue-period responsiveness and are shown as summed statistically significant main, two-way, and three-way spike-per-puff effects in Figure 4G. First, the on-task state 1 increased the puff dependence of cluster 3 from 0.58 ± 0.04 spikes/puff (mean ± SD) by an additional 0.23 ± 0.09 spikes/puff (*p* = 0.014) to a total of 0.81 ± 0.10 spikes/puff. Second, the past-choice-focused state 2 reduced the puff dependence of the outcome-dependent cluster 1 from 0.65 ± 0.03 spikes/puff by 0.23 ± 0.12 spikes/puff (*p* = 0.049) to 0.42 ± 0.12 spikes/puff. In short, the current-evidence-focused state 1 showed enhanced representation of current evidence compared with the previous-choice-focused state 2 in specific functional clusters (3 and 1) totaling 21% + 7% = 28% of dendrites.

### The on-task state suppresses complex-spike responses to trial outcomes

Complex spikes also fired in the 800 ms after the decision lick, a point during head-fixed tasks when a correct/incorrect choice leads to a reward/no reward. To obtain fit coefficients in units of effect size, we fitted an LME model to *Z*_decision_, the *Z*-scored number of dendritic calcium transients during the decision period (1.08 ± 1.05 transients, mean ± SD), as a function of behavioral state, dendrite cluster, and error-vs.-correct outcome. We included two-way and three-way interaction effects as well as random effects by mouse and dendrite.

This mixed-effects model revealed that outcome-period firing *Z*_decision_ varied strongly by state (*F*_(2, 17.304)_ = 22.33, *p* = 1.6 × 10^−5^, ANOVA marginal test, Satterthwaite criterion), outcome (*F*_(1, 67.219)_ = 18.273, *p* = 6.2 × 10^−5^), and cluster (*F*_(6, 26.3)_ = 52.747, *p* < 10^−12^). Behavioral state had strong effects on cluster-dependent activity (state × cluster, *F*_(12, 109.55)_ = 9.179, *p* = 5.2 × 10^−12^; state × cluster × outcome, *F*_(12, 4843.4)_ = 5.2447, *p* = 7.6 × 10^−9^), revealing that state regulated the outcome-dependent distribution of firing across clusters.

The largest main effects arose from behavioral state. Compared with state 3, the on-task state 1 was associated with a 0.61 ± 0.10 smaller response (*t* = 6.19, *p* = 6.2 × 10^−10^), and the past-trials state 2 showed a 0.39 ± 0.12 smaller response (*t* = 3.38, *p* = 7.3 × 10^−4^). These effects were partially counteracted by significant state × cluster effects for clusters 1, 2, and 3 (median increase of 0.27). Every one of the effects was larger than the main effect of decision error, an increase of 0.17 ± 0.04 (*t* = 4.27, *p* = 1.9 × 10^−5^). Thus, the overall trend (Figure 4H) was one in which during the outcome period, Purkinje cells produced most complex spikes in state 3, fewer in state 2, and least in state 1, consistent with the concept of complex spikes as a reporter of unexpected events. This trend was strongest in clusters 4 (strong response to cues), 5 (error trials), and 6 (tone).

In error trials, increased complex-spike responses were both cluster specific (cluster × error) and state specific (state × cluster × error, but not state × error). The tendency of mice to produce more spikes in state 3 was present in five out of the six clusters, the only exception being cluster 3, a puff-sensitive cluster. Overall, state 3 showed a response of 0.43 more than state 1, well over one-third of a standard deviation. In short, the encoding of decision errors via complex spikes was highly state dependent, and the majority of spikes occurred when mice were off-task.

### *L7-Tsc1* mutant mice have enhanced learning capabilities

**Figure 5.**
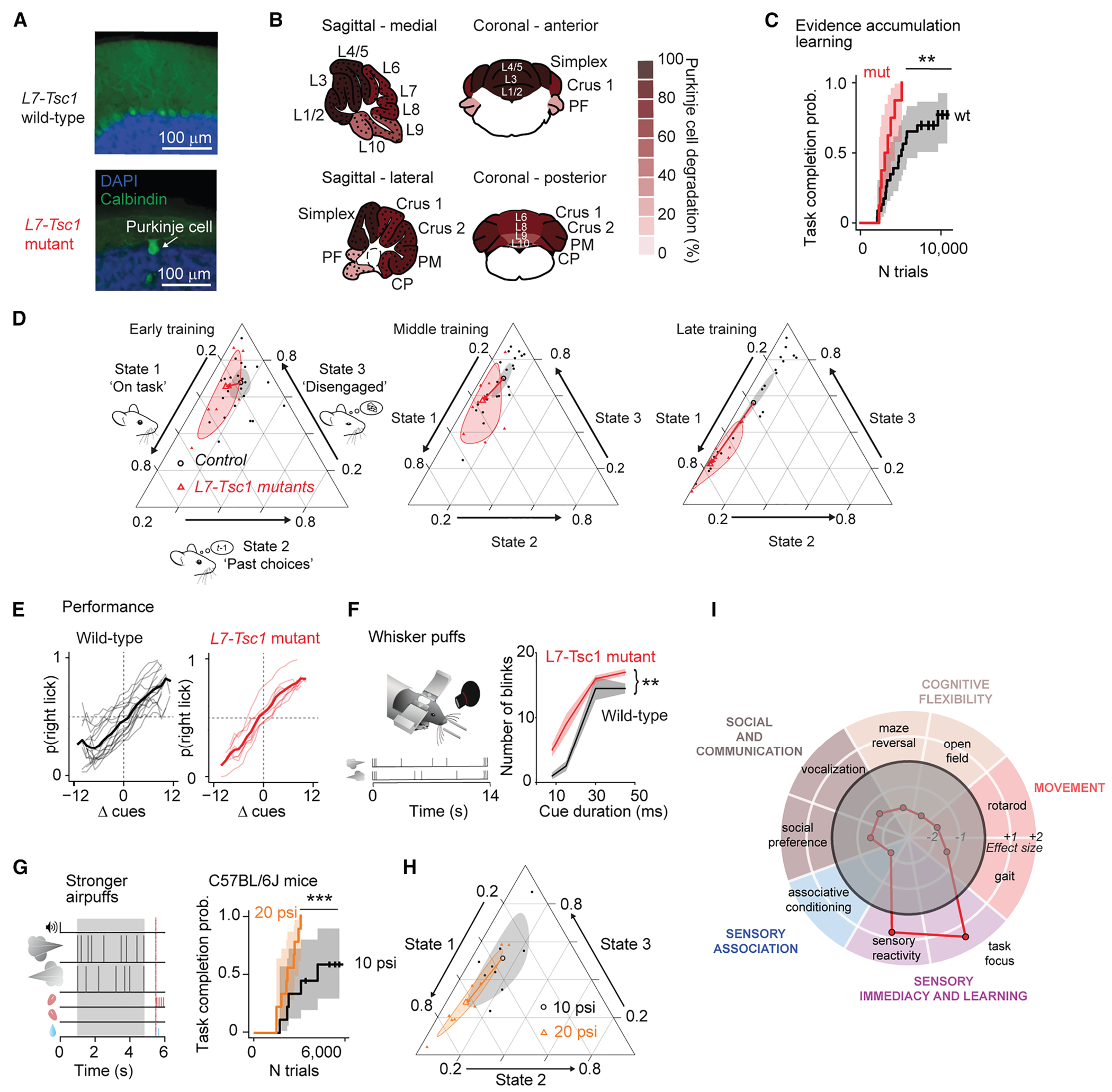
Cerebellar-impaired *L7-Tsc1* mutant mice show enhanced learning of an evidence-accumulation, decision-making task (A) *L7-Tsc1* mutant mice have reduced numbers of Purkinje cells in the cerebellar cortex. Scale bars, 100 μm. (B) Schematic of sagittal and coronal views of the cerebellum with quantification of Purkinje cell loss averaged over four *L7-Tsc1* mutant mice at 5–6 months old for each cerebellar lobule, normalized to three wild-type littermates. (C) Kaplan-Meier estimator of probability of reaching the final level of task training for *L7-Tsc1* mutant mice (log-rank test, **p ≤ 0.01). Shaded areas in Kaplan-Meier curves represent 95% CIs. (D) Ternary plots for the composition of latent states with 95% confidence bands (shaded regions) around the compositional mean (open symbols) for *L7-Tsc1* mutant mice and control mice (wild-type littermates). Each data point represents one mouse. The 95% confidence regions are based on an assumption of normality for compositions. Arrows indicate the shift from the average of the control group to the average of the *L7-Tsc1* mutant mice. (E) Psychometric performance curves in mice who recently reached the final level show no detectable change in bias, slope, or lapse rate. Thick lines indicate averages; thin lines are data from individual animals. Data from all eight *L7-Tsc1* mutant mice are included as well as 17 out of 23 control mice (six control animals did not reach the final version of the task). (F) Left: sensory sensitivity test with bilateral and unilateral whisker puffs in wild-type C57BL/6J mice. Right: median number of blinks in response to puffs of different durations for *L7-Tsc1* mutant mice (*n* = 16) and wild-type littermates (*n* = 7; two-way ANOVA, **p ≤ 0.01). Shaded areas indicate the estimated SEM. (G) Increased salience through stronger puffs leads to enhanced learning (log-rank test, ***p ≤ 0.001). Kaplan-Meier estimator of task completion for animals receiving standard (10 psi, nine mice) or stronger (20 psi, nine mice) puffs. Shaded areas represent 95% CIs. (H) Ternary compositional plot of latent states with 95% confidence bands (shaded regions) around the compositional mean (open symbols) at middle levels of training. The arrow indicates the shift from the 10-psi average to the group receiving stronger airpuffs. (I) *L7-Tsc1* mutant mice express an island of enhanced sensory reactivity and task focus amid a variety of impairments. Each dot represents an estimated effect size (Cohen’s *d*) for the behavior of *L7-Tsc1* mutant mice compared to their wild-type littermates. The thick circle indicates typical behavior (effect size 0). Results are based on data presented in this paper and from Tsai et al.,^18^ Kloth et al.,^17^ and Klibaite et al.^29^

To test the contributions of cerebellum-specific disruptions to learning in a model of neurodevelopmental disorder, we used *L7-Tsc1*, a mouse model with Purkinje-cell-specific knockout of tuberous sclerosis 1 (Tsc1) with reduced numbers of Purkinje cells (Figures 5A and 5B) that displays autism-like features.^18^

We were surprised to find that *L7-Tsc1* mutant mice showed enhanced learning capabilities, successfully reaching the final level of training almost twice as quickly as wild-type littermates (Figure 5C, mutant median 3,211 trials in eight mice compared with wild-type median 5,106 trials in 23 mice; *χ*^2^_(1)_ = 7.11, *p* = 0.007, log-rank test; for individual mouse learning curves; see Figure S3A). Acceleration of learning did not vary detectably across training level (number of trials to reach criterion, two-way ANOVA, level × genotype interaction, *F*_(2)_ = 1.23, *p* = 0.30, early [0, 1, and 2] vs. middle [3 and 4] vs. late training levels [5 and 6] comparison; see also Figure S3B). Learning can depend on sex, age, or stress level,^30^ but in our case there was no detectable difference in the number of trials by sex, age at start of training, or corticosterone level (Figure S3C; *F*_(3, 18)_ = 0.29, *p* = 0.83, multivariate ANOVA).

Once animals had reached an expert stage (level 7), in the first few sessions there was no difference in overall performance between *L7-Tsc1* mutant mice and their wild-type littermates (no significant difference in percentage of correct trials: *p* = 0.53, Welch’s *t* test, one-tailed; no significant difference in bias [*t*_(24)_ = 0.81, *p* = 0.43], slope [*t*_(24)_ = 0.10, *p* = 0.92], or lapse rate [*t*_(24)_ = 1.35, *p* = 0.19], two-tailed Student’s *t* tests of the psychometric curves). Mutant mice also did not differ from wild types in the number of licks per trial, either in correct trials (*p* = 0.45, two-tailed *t* test) or incorrect trials (*p* = 0.23). In short, final performance was comparable to that of animals that had been trained extensively at the same or a similar task.^19–21^

### Mutants stay on-task and in the present

Using our task-state analysis, we found that compared with wild-type mice, *L7-Tsc1* mutant mice had higher state 1 occupancy at later stages of training (Figure 5D). Compositional analysis of state occupancies and multivariate ANOVA on the log ratio state 2/state 1 revealed significant results for wild-type vs. *L7-Tsc1* mutant mice (*F*_(1)_ = 18.8, *p* < 0.001) and for training level (*F*_(2)_ = 24.5, *p* < 0.001). Post hoc comparisons using the Tukey HSD test indicated a significant result for later stages of training (levels 5–6, *p* = 0.006) but not for early (levels 0–2, *p* = 0.49) or middle stages of training (levels 3–4, *p* = 0.29). Multivariate ANOVA on the log ratio state 3/state 1 yielded a trend toward significance for wild-type vs. *L7-Tsc1* mutant mice (*F*_(1)_ = 3.6, *p* = 0.06) and no significant effect for training level (*F*_(2)_ = 1.9, *p* = 0.15). The shape of the psychometric curves of *L7-Tsc1* mutant mice in each state was similar to that of their wild-type littermates (Figures 5E, S5A, and S5B).

### Increased sensory intensity is sufficient to accelerate learning

In several mouse models, a variety of motor and nonmotor phenotypes have been shown to be regulated by enhanced sensory salience,^31^ raising the possibility that enhanced saliency of sensory events might also lead to accelerated learning in our task. We therefore measured sensory reactivity before training.

Naive *L7-Tsc1* mutant mice showed enhanced blink responses to individual airpuffs (Figure 5F) as well as to auditory stimuli (Figures S4A and S4B), indicative of altered sensory processing. In response to puffs of different durations (Figure 5F), *L7-Tsc1* mutant mice (*n* = 16) blinked more times than wild-type littermates (*n* = 7; *F*_(1)_ = 7.44, *p* = 0.008; no interaction with puff dependence [two-way ANOVA, *F* = 0.98, *p* = 0.4]). Responses to nose taps in mutants also tended to be longer in duration (Figures S4E–S4G). Therefore, *L7-Tsc1* mutant mice are hypersensitive across at least two sensory modalities.

However, we found that in delayed tactile startle conditioning (DTSC), a form of cerebellum-dependent associative learning that uses the nose tap as an unconditioned stimulus,^32–34^ mutant mice were slower, not faster, to learn (Figure S4C; mutant mice, median 1,000 trials to criterion in five mice compared with wild-type littermates, 500 trials in five mice: *χ*^2^_(1)_ = 9.70, *p* = 0.0018, log-rank test). After seven sessions, *L7-Tsc1* mutant mice had fewer trials with a conditioned response (CR) than wild-type lit-termates (Figure S4D; wild-type, CR in 62% ± 12% of trials; mutant mice, CR in 28% ± 16% of trials; *t*_(9)_ = 3.4, *p* = 0.009, two-tailed Student’s *t* test). Together with our finding that sensorimotor reflexes were unaffected by cue-locked optogenetic stimulation, these results suggest that the acceleration of evidence-accumulation learning from cerebellar perturbation, including in *L7-Tsc1* mice, might arise from processing steps outside of direct reflex pathways or cerebellar plasticity.

To test whether increased sensory intensity would be sufficient to accelerate learning, we increased the intensity of airpuffs from 10 psi to 20 psi in wild-type C57BL/6J mice. Stronger puffs led to faster learning (Figure 5G): 20-psi puffs (nine mice, median 2,225 trials) led to completion of training in fewer trials than with standard 10-psi puffs (nine mice, median 4,275 trials; *χ*^2^_(1)_ = 7.11, *p* = 0.00047, log-rank test), a degree of acceleration that was similar to that seen in *L7-Tsc1* mutant mice. The shape of the psychometric curves in trained mice was similar whether animals received 10-psi or 20-psi airpuffs (Figures S5C and S5D). This result shows that naturally occurring sensory reactivity is sufficient to accelerate learning of the evidence-accumulation task.

Stronger puffs also shifted behavior during learning toward the on-task state. The log ratio state 2/state 1 increased with training level (two-way ANOVA, *F*_(1)_ = 44.2, *p* < 0.001) and trended toward greater values at 20 psi (*F*_(1)_ = 3.5, *p* = 0.07). Post hoc comparisons using the Tukey HSD test indicated a trend toward significance for middle stages of training (levels 3–4, the earliest levels at which the manipulation occurred, *p* = 0.10; Figure 5H) but not for later stages of training (levels 5–6, *p* = 0.99). The log ratio state 3/state 1 was not different for either group (*F*_(1)_ = 1.4, *p* = 0.24) or training level (*F*_(1)_ = 0.8, *p* = 0.37). In summary, more intense puff stimulation was able to replicate a constellation of *L7-Tsc1* phenotypes: blinking more, staying on task, and learning faster. Taken together, these findings suggest that *L7-Tsc1* mice show functional consequences that can be mimicked by increased stimulus intensity.

From previous work, *L7-Tsc1* mutant mice are known to show perseveration and deficits in gait and social interactions as well as deficits in relatively simple motor learning on the accelerating rotarod.^17,18^ Taken together with the convergence of impaired associative motor learning, increased sensory sensitivity, and accelerated task learning (Figure 5I), these traits echo a particular feature of ASD, the association of sensory deficits with task overfocus, suggesting that this co-occurrence of traits could be explained by a cerebellum-based mechanism for increased salience and perseveration through abnormal sensory processing.

### Optogenetic boosting enhances complex-spike rebound and neocortical firing

**Figure 6.**
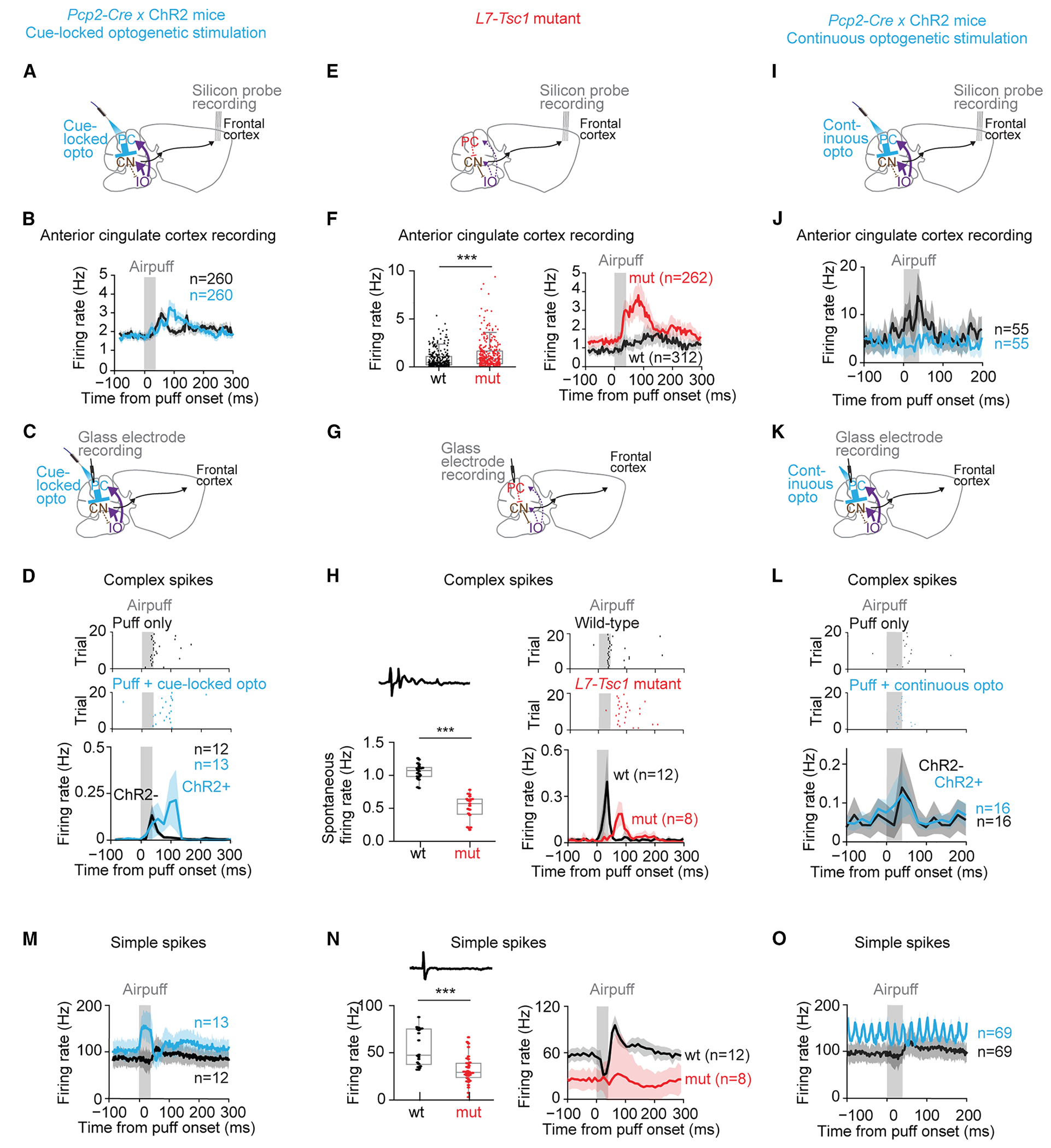
Altered whisker-puff responses in naive mice with genetic or optogenetic manipulation of Purkinje cells (A) Schematic of silicon probe recording site in forebrain from mice receiving cue-locked optogenetic stimulation of crus I. (B) Average firing rates in anterior cingulate cortex. The addition of optogenetic stimulation resulted in time-shifted responses to airpuffs. (C) Cerebellar recording site in the whisker puffs + cue-locked optogenetic experiment. (D) Example raster plots of Purkinje cell complex spikes during 20 trials with only a whisker puff (top) or with a whisker puff paired with cue-locked optogenetic stimulation (middle). Bottom: Purkinje cell complex-spike recordings (average data from four mice) show that pairing airpuffs with optogenetic stimuli led to a shift in the time course of activity toward later times after airpuff onset. (E) Schematic of silicon probe recording site in forebrain from *L7-Tsc1* mutant mice. (F) Left: increased spontaneous in vivo firing rates in the anterior cingulate cortex in *L7-Tsc1* mutant mice (two-tailed Student’s t test, ***p ≤ 0.001). Right: average firing rates in anterior cingulate cortex in response to an airpuff to the whiskers (four mutant and five wild-type mice). Responses varied somewhat by genetic background (compare with B and J). (G) Cerebellar recording site from *L7-Tsc1* mutant mice. (H) Left: reduced spontaneous *in vivo* firing rates of complex spikes in *L7-Tsc1* mutant mice (two-tailed Student’s t test, ***p ≤ 0.001). Above the plot is an example waveform of a complex spike; total duration of the example trace is 15 ms. Right: example raster plots of Purkinje cell complex spikes during 20 trials from one wild-type animal (top) and one *L7-Tsc1* mutant animal (bottom). Bottom right: Delayed time course of the airpuff evoked response of complex spikes compared with wild-type littermates. (I) Schematic of silicon probe recording site in forebrain from mice receiving continuous bilateral optogenetic stimulation of crus I. (J) Average firing rates in anterior cingulate cortex. The addition of optogenetic stimulation reduced responses to airpuffs. (K) Cerebellar recording site in the whisker puffs + continuous optogenetic experiment. (L) Example raster plots of Purkinje cell complex spikes during 20 trials with only a whisker puff (top) or with a whisker puff paired with continuous optogenetic stimulation (middle). Bottom: Purkinje cell complex-spike recordings show that pairing airpuffs with optogenetic stimuli led to a shift in the time course of activity toward later times after airpuff onset. (M) Simple spikes in the Purkinje cells stimulated with cue-locked optogenetic activation led to an increased firing rate without time shift. (N) Left: reduced spontaneous *in vivo* firing rates of simple spikes in *L7-Tsc1* mutant mice (two-tailed Student’s t test, ***p ≤ 0.001). Above the plot is an example waveform of a simple spike; total duration of the example trace is 15 ms. Right: delayed and smaller simple-spike response in mutant mice compared with wild-type littermates. (O) Simple spikes in the Purkinje cells stimulated with continuous bilateral optogenetic activation led to an increased overall firing rate without a specific response to the airpuff. In (B), (D), (F), (H), (J), (L), (M), (N), and (O) shaded areas represent 95% confidence intervals.

Influences of cerebellum are conveyed via long-range pathways that project throughout thalamus and neocortex,^35^ including two associative regions implicated in decision-making^36–38^ that receive substantial disynaptic input from crus I,^35^ anterior cingulate, and anterolateral motor cortex. Performance of an evidence-accumulation task depends on these and a variety of other neocortical regions.^39^ We performed *in vivo* silicon-probe electrophysiological recordings from these and other neocortical regions in naive, awake ChR2-expressing and *L7-Tsc1* mice (Figures 6 and S6).

In ChR2-expressing mice (Figure 6A), we found that single airpuff cues evoked transient firing increases in the anterior cingulate cortex whose onset coincided with the end of the stimulus (Figure 6B, three mice). The addition of cue-locked optogenetic stimulation to crus I resulted in a delayed response in anterior cingulate cortex (Figure 6B), with a larger response in the 50–100 ms following airpuff onset (airpuffs + optogenetic stimuli [airpuffs + opto] = 200 ± 390, airpuffs only = 151 ± 323; *t*_(259)_ = −4.3, *p* < 0.0001, paired *t* test) and a reduced response in the first 50 ms (relative to baseline rate pre-stimulus: airpuffs + opto mean area under the curve [AUC] = 124 ± 212, airpuffs only = 167 ± 338; *t*_(259)_ = 4.6, *p* < 0.0001, paired *t* test). We also observed an enhancement in anterolateral motor region (Figure S7B) activity, but not in the barrel field of the primary somatosensory cortex (Figure S7C). This enhancement observed in the anterolateral motor cortex seemed to be driven by the offset of the optogenetic stimulus, as was the case when we paired a 40-ms airpuff with a 250-ms optogenetic stimulus (Figure S8A). Similar to the 40-ms optogenetic stimulus, a 250-ms optogenetic stimulus also did not elicit a change in firing rate in the somatosensory cortex (Figure S8B). These observations reveal the ability of cerebellar optogenetic stimuli to enhance neocortical sensory responses.

Delayed excitatory drive to neocortex might arise from complex-spike firing, which is capable of post-stimulus rebound effects via disinhibitory feedback by nucleo-olivary paths.^40–42^ In separate experiments, we recorded from Purkinje cells in crus I (Figure 6C) and found that airpuffs triggered complex spikes (Figure 6D) which, like neocortical responses, occurred at the time of stimulus offset. Pairing with cue-locked optogenetic stimulation led these complex spikes to be delayed further (Figure 6D), with a larger response in the 50–100 ms following puff onset (airpuffs + opto = 1.8 ± 0.3, airpuffs only = 0.7 ± 0.4; *t*(24) = − 7.5, *p* < 0.0001), an accompanying decrease in the deep nuclear response (airpuffs + opto = 10 ± 6, airpuffs only = 17 ± 7, *t*_(16)_ = 2.3, *p* = 0.03; Figure S7A), and a smaller complex-spike response during the first 50 ms (airpuffs + opto mean AUC = 0.4 ± 0.3, airpuffs only = 1.0 ± 0.8; *t*_(24)_ = 2.5, *p* = 0.02, two-tailed Student’s *t* test). Finally, airpuff-evoked complex spikes reached 90% of the maximum firing rate at 25 ± 8 ms (latency from airpuff to start of firing, 13 ± 7 ms) followed by a significant gap in time by anterior cingulate firing at 137 ± 83 ms (latency from airpuff to start of firing, 72 ± 70 ms) (difference in peak firing time: *t*_(271)_ = − 4.5, *p* = 0.00001; latency: *t*_(271)_ = − 2.8, *p* = 0.006), consistent with a complex-spike-driven mechanism.

### Mutant mice show enhanced complex-spike rebound and neocortical firing

We also recorded from awake *L7-Tsc1* mutant mice (Figure 6E). *L7-Tsc1* mutant mice had higher spontaneous *in vivo* firing rates in the anterior cingulate cortex than wild-type littermates (Figure 6F; wild-type mean 0.84 Hz in 312 cells from five mice, mutant mean 1.34 Hz in 262 cells from four mice; *t*_(573)_ = 3.6, *p* = 0.0004), consistent with our past finding that acute chemogenetic suppression of crus I Purkinje cell activity leads to increased activity in anterior cingulate cortex.^43^ We furthermore found that airpuff-evoked responses were considerably larger in mutant mice than in their wild-type littermates (Figure 6F; AUC relative to baseline rate pre-stimulus: 678 ± 1,109 in mutant mice, compared to 398 ± 646 in wild-type mice; *t*_(573)_ = − 3.6, *p* = 0.0003, indicative of enhanced cerebellar drive), suggesting that either loss or altered function of Purkinje cells could exert long-distance effects on anterior cingulate cortex activity.

Despite the fragility of surviving *Tsc1*^−/−^ Purkinje cells,^18^ we were able to record from them electrophysiologically (Figure 6G). *Tsc*^−/−^ Purkinje cells showed lower firing *in vivo* with reduced complex-spike activity (Figure 6H, wild-type mice 1.05 ± 0.12 Hz in 20 cells, mutants 0.51 ± 0.19 Hz in 19 cells from four mice; *t*_(38)_ = 10.55, *p* = 1.1 × 10^−12^, two-tailed Student’s *t* test), consistent with *ex vivo* observations.^18^

In mutant mice, airpuffs activated complex spikes after a latency of 53 ± 7 ms (mean ± SD), longer than the 13 ± 7 ms seen in wild-type littermates (Figure 6H; *t*_(19)_ = − 10.7, *p* < 0.0001). The time course of responses was delayed: the mean AUC was higher for mutants during the 50–100 ms following airpuff onset (relative to baseline rate pre-stimulus: mutant mice AUC = 5.1 ± 2.0, wild-type littermates’ AUC = 0.8 ± 0.7; *t*_(19)_ = − 6.3, *p* < 0.0001), while the AUC in the 0- to 50-ms interval was higher for wild-type mice (mutant mice AUC = 1.1 ± 0.5, wild-type AUC = 7.1 ± 2.0; *t*_(19)_ = 7.89, *p* < 0.0001). Thus, like cue-locked optogenetically stimulated mice, *L7-Tsc1* mice show a delayed increase in airpuff-evoked complex spiking. Response timing between cerebellum and forebrain was more closely spaced than in cue-locked optogenetic stimulation experiments, with complex spikes reaching 90% of maximum firing rate at 73 ± 5 ms (latency, 53 ± 7 ms) followed by anterior cingulate at 106 ± 78 ms (latency, 46 ± 49 ms; peak timing: *t*_(316)_ = − 1.1, *p* = 0.26; latency: *t*_(316)_ = 0.35, *p* = 0.73). We also observed an enhancement in the anterolateral motor region (Figure S7E), but not in the barrel field of the primary somatosensory cortex (Figure S7F). Thus, as with cue-locked optogenetic stimulation, Tsc1-knockout-driven increases in cerebellar complex-spike output can cause increases in, as well as changes in timing of, neocortical activity.

In contrast to cue-locked optogenetic stimulation and Tsc1^−/−^ mice, continuous stimulation in naive ChR2^+^ mice (Figures 6I and 6K) did not change whisker puff responses in forebrain areas (Figures 6J, S7H, and S7I), complex-spike firing (Figures 6L and 6O), or deep nuclei (Figure S7G).

### Enhancements of learning are not correlated with simple-spike perturbations

So far we have shown that learning can be augmented by enhancing Purkinje cell activity during sensory stimulation, either by cue-locked optogenetic stimulation or via *Tsc1* knockout. Both of these perturbations generate similar post-stimulus increases in complex-spike timing and neocortical activity. However, they also affect simple-spike firing, which likewise affects processing outside the cerebellum.

Cue-locked and continuous optogenetic stimulation both show increased simple-spike activity but, unlike cue-locked stimulation, continuous stimulation did not enhance simple-spike responses to individual sensory cues (Figures 6M and 6O). In cue-locked stimulation, in the first 50 ms following airpuff onset, more simple spikes fired under the airpuffs + opto condition (airpuffs + opto mean AUC = 26 ± 9, airpuffs only = 14 ± 5; *t*_(24)_ = − 3.7, *p* = 0.001), but there was little measurable difference in the next 50 ms (airpuffs + opto = 14 ± 5, airpuffs only = 16 ± 5; *t*_(24)_ = 1.18, *p* = 0.25).

In *L7-Tsc1* mice, both spontaneous and airpuff-evoked simple-spike responses were reduced (for spontaneous responses: wild-type mice had a spontaneous simple-spike firing rate of 55.3 ± 18.8 Hz [mean ± SD] in 20 cells from five mice, whereas mutant littermates had a spontaneous simple-spike firing rate of 31.9 ± 14.3 Hz in 34 cells from four mice, *t*_(53)_ = 5.06, *p* = 5.5 × 10^−6^; for evoked responses: wild-type mean AUC = 191 ± 27, mutant mean AUC = 71 ± 85; *t*_(19)_ = 4.3, *p* = 0.0004), and evoked responses had a later onset (Figure 6L; latency in wild-type mice 11 ± 8 ms [mean ± SD]. mutant mice 29 ± 15 ms; *t*_(19)_ = − 3.3, *p* = 0.004). As expected, this decreased simple-spike response also led to disinhibition of negative feedback from deep nuclei (Figure S7D) onto inferior olivary neurons.^41,42^

In summary, conditions that accelerate learning are associated with increased simple spiking during airpuffs (cue-locked optogenetic) or reduced and delayed simple spiking (*L7-Tsc1*^−/−^ ), while lack of accelerated learning is associated with an increase in the rate of cue-independent simple spiking.

### During training, cerebellar activation enhances anterior cingulate responses to evidence

**Figure 7.**
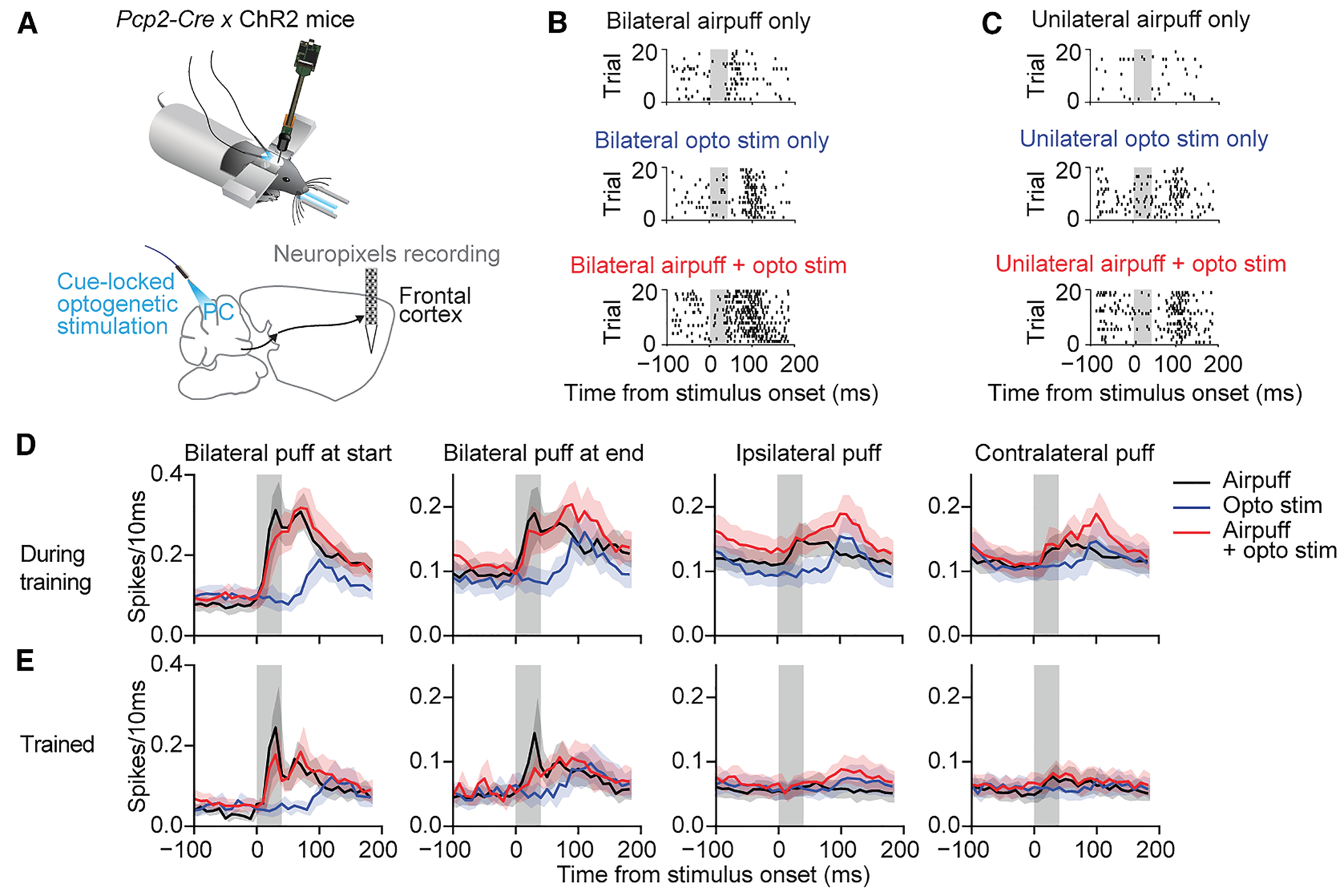
Neural responses in anterior cingulate cortex to optogenetic stimulation of the cerebellum and whisker-puff stimuli during the evidence-accumulation task (A) Execution of the evidence-accumulation task during acute Neuropixels recordings and bilateral optogenetic stimulation of the cerebellum (top) and forebrain recording site in addition to cue-locked optogenetic stimulation of Purkinje cells (bottom) in *Pcp2-Cre* × ChR2 mice. (B) Spike rasters from an example single unit in anterior cingulate cortex during training in response to bilateral stimuli: airpuffs only (top), optogenetic stimulation only (middle), or both (bottom). (C) Same as (B), but for unilateral stimuli. (D) Average firing probability in response to a stimulus (black, airpuffs only; blue, optogenetic stimulation only; red, airpuffs combined with optogenetic stimulation) during levels 3–5 of training (*n* = 165 units from three recordings from three mice). (E) Same as (D), but for highly trained mice at level 7 or late stage of level 6 (*n* = 98 units from three recordings from two mice). Shaded areas in (D) and (E) represent 95% CIs.

With some effort (Figures 7A and S6C) it was possible to make acute Neuropixels recordings while also delivering optogenetic stimuli as mice performed evidence accumulation. In *Pcp2-Cre* × ChR2 mice undergoing level 3–5 training, we identified 165 units in anterior cingulate cortex that responded to bilateral whisker stimulation marking the start of the trial (Figure 7B), which we then used to probe cerebellum-forebrain interactions. These units also responded to unilateral evidence puffs presented on either side (Figure 7C) as well as to cerebellar optogenetic stimuli (with a delay of 20–30 ms; Figures 7B and 7C).

During a 200-ms post-stimulus period, pairing optogenetic stimuli with evidence airpuffs evoked a larger neural response than evidence airpuffs alone (Figure 7D) (ipsilateral: 200-ms integrated response 27.8 ± 24.7 for paired stimulation, 23.1 ± 20.6 for airpuffs alone, *p* < 0.001; contralateral: 200-ms integrated response 26.4 ± 25.8 for paired stimulation, 22.8 ± 20.4 for airpuffs alone, *p* < 0.001). This enhancement did not occur for the bilateral airpuffs that marked the start or the end of each trial. Thus, cue-locked cerebellar optogenetic stimuli specifically enhance a forebrain effect of evidence airpuffs.

The completion of training (level 7 and late-stage level 6, *n* = 98 units) was associated with smaller responses in anterior cingulate cortex units to both unilateral and bilateral airpuffs (Figure 7E; two-way ANOVA, main effect of training vs. completed training with puff/opto/puff + opto as the second variate: start-of-trial bilateral puffs, *F*_(1)_ = 54.3, *p* < 10^−12^; end-of-trial bilateral puffs, *F*_(1)_ = 32.3, *p* = 2.0 × 10^−8^; ipsilateral puffs, *F*_(1)_ = 48.9, *p* = 7.4 × 10^−12^; contralateral puffs, *F*_(1)_ = 50.8, *p* = 3.0 × 10^−12^; no interaction effects for any stimulus, *p* > 0.1). Taken together, these results demonstrate that during training, cerebellar stimulation enhances anterior cingulate cortex response to evidence airpuffs, and this enhancement diminishes as training saturates.

## DISCUSSION

Our experiments support the idea that cerebellar complex-spike output can accelerate nonmotor learning, which may be explained by increased on-task focus and modulation of brainwide responsivity to task variables, especially sensory cues. Based on anatomical evidence, these cerebellar influences are likely to be transmitted to neocortical structures via major paths through thalamus and other midbrain structures^35,44^ to support delayed activation and enhanced forebrain responses.

In this work, we have demonstrated the ability of optogenetic stimulation to reinforce task-related learning mechanisms. Delivering optogenetic stimuli in a cue-locked manner had two effects. First, if given during the training period, cue-locked stimuli could accelerate learning. Second, after training using cue-locked optogenetic stimuli, the same optogenetic stimuli were sufficient on their own to bias the mouse’s lick choices in the direction of stimulation. These findings show that it is possible for cue-locked optogenetic stimulation to boost the effects of sensory stimuli for both learning and the evidence-accumulation process.

Our results are consistent with the idea that complex spikes can enhance the salience of sensory stimuli and that this enhancement can be transmitted to frontal cortex, including anterior cingulate, using rebound-firing-based mechanisms in the deep nuclei. These signals are larger during the learning of a complex task than after learning. They can account for the accelerated learning in *L7-Tsc1* mice because those mice, while impaired in their Purkinje cell function, still produce complex spikes but with a delay after the stimulus. We base this explanation on our comparison of the consequences of *L7-Tsc1* knockout on thalamocortical activity with the effects of focal crus I-specific cue-locked optogenetic enhancement. During a long period following each stimulus, both models led to enhancements in complex spikes and nuclear activity, enhancements in anterior cingulate cortex/anterior lateral motor cortex firing, and an alteration in the timing of all responses. Strong inhibition of cerebellar nuclear neurons is known to lead to post-inhibitory rebound,^16,45^ especially in lateral nuclei,^15^ which is transmitted to thalamus and inferior olive. Thus, our cue-locked optogenetic perturbation may replicate the *L7-Tsc1* condition’s effect on post-stimulus thalamocortical activity using a rebound deep nuclear mechanism through changes in timing, amplitude, or both.

Our work complements recent findings about roles for crus I in driving cognitive processing. Complex-spike activity in crus I can encode cue identity or perceptual choices, and this activity is coupled with anterior cingulate cortex under social but not nonsocial conditions,^46^ suggesting that information ascending from cerebellum is actively regulated. Indeed, crus I can tag salient moments such as on-goal events and perceptual choices.^47,48^ We note a striking parallel from motor learning, where complex spikes can instruct plasticity in primary somatosensory cortex.^49^ Furthermore, complex-spike responses to sensory stimuli have been shown to be reduced during specific phases of locomotion,^50^ indicative of a gating mechanism that allows relevant stimuli through. Our results suggest that such an instructive mechanism may act via rebound firing in the deep nuclei to entrain forebrain mechanisms, including systems that support evidence accumulation and decision-making. We suggest that sensory gating, salience, and task state may be different facets of a shared cerebellar mechanism for regulating behavior.

Additional mechanisms not shared between the two perturbations might also hypothetically account for our findings. For example, although recorded Purkinje cells in *L7-Tsc1* mice showed an increase in airpuff-evoked simple-spike responses (Figure 6N), they also showed reductions in the number of Purkinje cells (Figures 1A and 1B). The reduced overall Purkinje cell drive in *L7-Tsc1* mutant mice could account for our observation that both baseline and evoked deep nuclear output were increased. At a later stage of processing, the effects of increased deep nuclear output are transmitted to forebrain via nucleothalamic synapses, which have recently been found to be strengthened in *L7-Tsc1* mice.^51^ These loci provide additional mechanisms for cerebellar alterations in *L7-Tsc1* mice to cause hyper-responsiveness in forebrain.

### Limitations of the study

Although anatomical evidence supports a projection from cerebellar crus I to the anterior cingulate cortex, the necessity of this specific pathway for the observed learning enhancement has not been directly tested. The anterior cingulate cortex should therefore be considered an example target of cerebellar influence among multiple neocortical regions known to contribute to evidence accumulation. Additionally, because cerebellar and anterior cingulate cortex recordings were conducted in separate animals rather than simultaneously, a direct trial-by-trial relationship between complex-spike activity and anterior cingulate cortex responses requires simultaneous multi-region recording or direct climbing-fiber manipulation. A second limitation concerns the interpretation of latent behavioral state 1 as reflecting heightened on-task focus, which is an inference from behavioral correlates rather than a direct measure of attention. Third, the learning acceleration observed in *L7-Tsc1* mice vs. cue-locked optogenetic stimulation in wild-type mice may operate through somewhat different complex-spike mechanisms, for instance timing vs. amplitude changes in cerebellar-cortical signaling. Finally, the distinction between altered sensory gain and attentional modulation is an important question for future investigation.

### The cerebellum and global coherence

The convergence of impaired associative motor learning, increased sensory sensitivity, and accelerated task learning in *L7-Tsc1* mice (Figure 5I) echoes traits found in ASD. ASD is associated with islands of enhanced function, including perceptual domains and technical and even artistic capacities.^52,53^ According to the weak central coherence account of ASD, these enhanced capacities can be explained by a detail-focused cognitive style in which individual perceptual features are emphasized. In the global coherence account of ASD, the capacity to extract global form and meaning is displaced by superiority on local or detail-focused processing.^52^ Our findings with cerebellar perturbations demonstrate one aspect of such processing, sensory hypersensitivity,^53^ and an association with accelerated capacity to learn a sensory-integration task. In mouse models, enhancement of peripheral sensory sensitivity leads to alterations in central brain circuitry including anterior cingulate cortex, as well as ASD-like motor and nonmotor phenotypes.^31,54^

In *L7-Tsc1* mutant mice, inhibition of the medial prefrontal cortex has previously been found to relieve social deficits and repetitive behaviors.^55^ Among the extensive neocortical targets of cerebellar projections is the parietal cortex, where, interestingly, silencing of activity was recently shown to improve performance in evidence accumulation by reducing reliance on past evidence.^56^ In addition, transcranial direct current stimulation of the right lateral posterior cerebellum in humans improves performance on a sentence-completion task as well as altering activity in multiple neocortical regions.^57^ Finally, manipulation of a crus I projection to the dentate cerebellar nucleus affects dopamine release in striatum,^58^ and reduced Purkinje cell activity can lead to enhanced post-inhibitory increases in cerebellar output to regulate the strength and timing of action.^59^ These routes provide ways for the cerebellum to influence forebrain function.

Our results show that cerebellar perturbation by itself can lead to one predicted feature of increased sensory salience, the hyper-reactivity of neocortical circuits.^60^ In this way, cerebellar disruption provides one neural substrate for a seemingly paradoxical phenomenon seen in ASD: broad disruption of cognitive and social function accompanied by high performance in specific skill domains.^52^ This combination of features is reminiscent of atypicalities of sensation and perception reported in ASD, which have been interpreted in terms of a broadening of Bayesian priors about the sensory world. Such “hypo-priors” can account for a tendency among autistic persons “to perceive the world more accurately rather than [be] modulated by prior experience.”^61^ Expressed over development, the action of hypo-priors on forebrain circuitry could lead to lasting changes in regions that regulate social and cognitive function.^12^ In this way, sensory learning from the early-life environment, driven by the cerebellum or any brain region, may play a critical role in the development of adaptive behavior.

## RESOURCE AVAILABILITY

### Lead contact

Requests for further information and resources should be directed to the lead contact, Samuel S.-H. Wang (sswang@princeton.edu).

### Materials availability

This study did not generate new unique reagents.

## STAR★METHODS

### EXPERIMENTAL MODEL AND STUDY PARTICIPANT DETAILS

Experimental procedures were approved by the Princeton University Institutional Animal Care and Use Committee (protocol 1943-19) and performed in accordance with the animal welfare guidelines of the National Institutes of Health and in line with the European Directive 2010/63/EU on the protection of animals used for experimental purposes.

Data came from 156 mice (males and females, 2–5 months of age at the start of experiments) of genotypes C57BL/6J (The Jackson Laboratory, Bar Harbor, ME, 40 animals), *Pcp2-Cre* for Purkinje cell specificity and Ai27D for channelrhodopsin-2 (38 animals *Pcp2-Cre* × Ai27D, acquired from The Jackson Laboratory, stock #010536 (RRID:IMSR_JAX:010536) and #012567 (RRID:IM-SR_JAX:012567), respectively), 3 *Pcp2-Cre* × Ai148 mice for Purkinje cell dendritic imaging (Ai148 line acquired from Hongkui Zeng, Allen Brain Institute), and *L7*^Cre^;*Tsc1^flox/flox^* mice (75 animals). To create these Purkinje cell specific *L7*^Cre^;*Tsc1^flox/flox^* mice, *Tsc1^flox/flox^* (*Tsc1^tm1Djk^*/J, The Jackson Laboratory stock #005680) mutant mice were crossed into L7-Cre mice (B6.129-Tg(Pcp2-cre)2Mpin/J, The Jackson Laboratory, stock #004146). Our control mice comprised all littermates arising from the breeding strategy with unaltered Tsc gene expression in the offspring. We included all such littermates: *Tsc1^flox/flox^* (9 mice for behavior, 9 mice for electrophysiology), *Tsc1*^*flox/*+^ (11 mice for behavior, 8 mice for electrophysiology), *L7^Cre^*;*Tsc1*^+*/*+^ (1 mouse for behavior, 1 mice for electrophysiology), and *Tsc1*^+*/*+^ (2 mice for behavior, 1 mice for electrophysiology). Learning rates in the evidence-accumulation task among these 4 control groups were not significantly different from one another (one-way ANOVA, F(3) = 0.078, *p* = 0.97). Control behavior and neurophysiological measures were therefore reported as a single group for the *L7-Tsc1* mice. Because of differences in learning rates across genotypes (i.e., C57BL/6J, *L7-Tsc1*, and *Pcp2-Cre* × *Ai27D*), we made comparisons with suitably matched controls with conditions matched as closely as possible. Experimenters were blinded to the genotypes of the mice for the duration of the behavioral experiments.

All mice were group-housed in reverse light cycle to promote maximal performance during behavioral testing, which took time during the day. For long-term behavioral experiments, mice were housed in darkness in an enrichment box containing bedding, houses, wheels (Igloo and Fast-Trac; blue, red, or amber; K3328/K3570/K3327/K3250/K3251; Bio-Serv; Flemington, NJ, USA), climbing chains, and play tubes during all experimental days. At other times, mice were housed in cages in the animal facility, in groups of 2–4 mice per cage. During experiments in which water intake was restricted, mice received 1.0–1.5 mL of filtered water per day plus half of a mini yogurt drop (F7577; Bio-Serv; Flemington, NJ, USA), and body weight and condition was monitored daily. Mice always had *ad libitum* access to food pellets.

### METHOD DETAILS

#### Surgical procedures

For all surgeries, mice were anesthetized with isoflurane (5% for induction, 1.0–2.5% for maintenance), and were given buprenorphine (0.1 mg/kg body weight) and Rimadyl (5 mg/kg body weight) after surgery and were given at least 5 days of recovery in their home cages before the start of experiments, except for acute *in vivo* electrophysiology experiments when the animals were allowed to recover for at least two hours between the craniotomy and the acute recordings.

For mouse *in vivo* imaging experiments, a 3-mm-diameter craniotomy was drilled over the left posterior hemispheric cerebellum of *Pcp2-Cre* × Ai148 mice. In all imaged mice, a window composed of a cannula (Ziggy’s Tubes and Wires, # 09R304-363, 16 S/S Hypo Tube, 9 R GA. 0.1470/0.1490′ OD × 0.1150/0.1200′ ID × 0.0197′ long) glued (Norland Optical Adhesive 71) to a glass coverslip (Warner Instruments 64–0720) was cemented atop the craniotomy, and then a custom-machined titanium headplate was cemented to the skull using dental cement (C and B Metabond, Parkell Inc.).

For optogenetic experiments, a headplate was implanted as described above, after which two ~500 μm diameter craniotomies were drilled over the cerebellum, one over each hemisphere, directly posterior to the lamboid suture and ~3.6 mm lateral to the midline in either direction. During optical fiber placement we identified crus I starting first with coordinates (posterior 0.5 mm and lateral 3.6 mm from lambda, referenced relative to a diagonal approach perpendicular to the bone) and then adjusting aim based on the appearance of blood vessels on the surface of the brain. In initial experiments, location in crus I was confirmed histologically. Ferrule implants were constructed with 400-μm-diameter optical fiber (Thorlabs FT400EMT) glued to 1.25-mm OD stainless steel ferrules (Precision Fiber Products MM-FER2007-304-4500) using epoxy (Precision Fiber Products PFP 353ND). Ferrules were positioned over each craniotomy with the fiber tip at the surface of the dura mater, and Vetbond (3 M) was applied surrounding the exposed fiber. Dental cement was then applied to secure the ferrule to the skull. Implants were cleaned before each behavior session using a fiber optic cleaning kit (Thorlabs CKF).

Optogenetic experiments were performed in *Pcp2-Cre* × ChR2 mice. Optogenetic experiments were not pursued in *L7-Tsc1* mutants due to difficulties in generating a viable triple-transgenic line (*L7-Cre* × *Tsc1* mutant × ChR2 mice) with healthy Purkinje cells suitable for targeted stimulation.

For *in vivo* electrophysiology, a headplate was implanted as described above, and a 2 mm craniotomy was drilled over the area of interest and the dura removed. For recordings from neocortex, the following stereotaxic coordinates were used: anterior cingulate cortex: ML 0–0.5 mm, AP 0.5–1.5 mm, DV 0.7–1.0 mm, anterolateral motor cortex: ML 1.5 mm, AP 2.5 mm, DV 0.7–1.0 mm, and barrel field of the primary somatosensory cortex: ML 2.5 to 3.5 mm, AP − 0.8 to − 1.8 mm, DV 0.6 to 1.5 mm. Two stainless steel screws for ground and reference wires (000–120 1/16 SL bind machine screws, Antrin Miniature Specialties) were inserted in the skull above the forebrain as far away from the craniotomy as possible. For cerebellar recordings, a small hole was drilled for a reference electrode in the interparietal bone at the midline. Craniotomies (0.5 mm by 1.0–1.5 mm) were made next to the intersection of the interparietal and occipital bones and for silicon probe recordings made as lateral as possible (ML 2.8 mm) to reach crus I, and for glass electrode recordings centered over the left and right lobule V and simplex, allowing a diagonal antero-posterior approach to reach crus I (see Figure S6A). Craniotomies were covered with Kwik-Cast silicone adhesive (World Precision Instruments) until the time of the recording.

#### Behavior experiments

Mice were trained to perform an evidence-accumulation decision-making task as described previously.^19,21^ The behavioral apparatuses were controlled by custom-written Python software as published previously^19^ (https://github.com/wanglabprinceton/accumulating_puffs). Animals were trained for 1.5–9.0 weeks, 7 days/week.

Briefly, head-fixed mice were seated in a tube for daily 1 h behavioral sessions consisting of 200–300 trials. In each trial, independent streams of randomly timed 40 ms airpuffs of 10 psi (unless otherwise indicated) with a minimum 200 ms interpuff interval were delivered to the left and right sides over the course of a 1.0–3.8-s cue period. Each trial begins and ends with a bilateral puff to mark the cue period.

After a delay period of 200–800 ms, lick ports were advanced into the reach of the animal, and animals received a 4 μL water reward when they licked to the side with the greater number of puffs. The animal’s decision was interpreted as the side licked first, regardless of subsequent licks. Anti-biasing procedures^19^ result in chance levels being <50%. To increase motivation, restriction of water intake started at least 5 days before the start of training and continued throughout the whole training period. Error trials included a buzzer sound and a prolonged inter-trial interval.

Animals went through different levels of training (levels 0–6) to reach the final version of the task (level 7). The very first time the animal is in the setup, they need to learn that if they touch the lick spouts with their tongue, they will receive a drop of water. Therefore, in level 0 only, any first lick of each trial is rewarded with a drop of water, regardless which side the animal licked on or which side was correct for that trial. In levels 1 and 2, guide puffs occur at a regular interval (2.5 Hz) only on the correct side after the cue period ends and until the animal makes a first lick. Guide puffs are meant to be a hint to the animal as to where the correct side is, and allow the animal to base their decision on the most recent airpuff only (see Figure S1 left). Mice automatically proceeded to the next level once they reached pre-defined performance criteria (see Table S1 for details of each level as well as the performance criteria). The time it took an animal to learn the task was defined as the total number of trials to reach level 7. Psychometric curves were fitted with the psychofit module.^62^

Light for optogenetic stimulation during the evidence-accumulation task was delivered as described previously.^21^ Cue-locked optogenetic cerebellar activation occurred unilaterally, at the same side and time at an airpuff, at an intensity of 1.5–5.0 mW for a duration of 40 ms (generated by Master-8, A.M.P.I.). Continuous optogenetic activation occurred bilaterally with 5-ms pulses at 50 Hz throughout the entire cue period, delay period, and ended upon first lick contact. When optogenetic activation was used to manipulate the learning rate, the optogenetic activation only started from level 3, and at every trial from then on. When optogenetic activation was used to manipulate performance in trained mice, light was on in 20% of trials. In this case, analysis compares light-off and light-on trials only from behavioral sessions in which light was delivered.

For the delay tactile startle conditioning (DTSC) task,^32,33^ mice learned to elicit a startle (backward) movement in response to an initially neutral conditioned stimulus (CS; 250 ms; 5 mm 395–400 nm UV Ultraviolet LED, EDGELEC) that was paired to co-terminate with a startle-eliciting unconditioned stimulus (US, 50 ms tactile stimulus on the nose by taping foam to the stepper motor shaft (High Torque Nema 17 Bipolar Stepper Motor 92oz.in/65Ncm 2.1 A Extruder Motor, Stepper Online); CS-US inter-stimulus interval, 200 ms). Task performance was defined as the fraction of trials with a conditioned response (CR) and completion of task training was defined as the generation of a response of at least 20 steps on the rotary encoder on at least 60% of trials.^33^

For sensory sensitivity tests, naive animals were head-fixed in a similar setup to the evidence-accumulation setup and received either whisker puffs or auditory cues. Animals were not trained nor expected to do anything in response to the sensory cues, and did not receive any rewards throughout the session. Animals received cues in sequences of in total 24 cues starting and ending with three cues with 200 ms inter-cue interval, and in between those, cues at random intervals (ranging from 0.8 to 3.0 s). Animals first received a sequence with cue durations of 8 ms, followed by sequences with longer cue durations (15, 30, and 45 ms for whisker puffs, and 15, 30, 45, 90, 180, 320, and 640 ms for auditory cues). Animals either received bilateral airpuffs to the whiskers at 20–25 psi, or auditory cues at 12 kHz. During sensitivity tests with whisker puffs, white noise was on in the background throughout the experiment. To determine eye blink responses, movies of the right side of their face and body were acquired using two USB cameras (Playstation Eye), modified by removal of infrared filters and encasings. Images were acquired at 30 Hz with 320 × 240 pixel resolution. Illumination was provided by an infrared LED array (Yr.seasons 48-LED Illuminator Light CCTV 850 nm IR Infrared Night Vision). Airpuffs were produced by activation of solenoids (NResearch, standard two-way normally closed isolation valve, 161T011) with input from an air source (ControlAir Type 850 Miniature Air Pressure Regulator). Air was delivered via two tubes custom-machined with uniform openings, and positioned parallel to one another, parallel to the anteroposterior axis of the animal, 10 mm apart mediolaterally and ~1 mm anterior to the nose of the animal. Auditory cues were delivered to the apparatus by a speaker (Sony Tweeter XS-H20S) mounted below the apparatus. Analysis of eye blinks was performed using FaceMap^63^ (https://github.com/MouseLand/facemap) with manual curation and further analysis in Python.

#### Two-photon imaging

The procedure for two-photon imaging was similar to Deverett et al.^19^ Briefly, imaging was done using a custom two-photon microscope (Sutter Instrument Company, movable objective microscope with resonant scanning) under separate computer control using the MATLAB software ScanImage 2015^68^ (Vidrio Technologies, RRID: SCR_014307). Excitation light was provided by a Mai Tai Sapphire laser (Spectra-Physics) at 920 nm. A 16× objective lens (Thorlabs, 16× Nikon CFI LWD Plan fluorite objective, 0.80 NA, 3.0 mm WD) was used with ultrasound gel (Sonigel, Mettler Electronics) as the immersion medium. Excitation power measured at the output of the objective lens ranged from 10 to 50 mW. Images were acquired at 28 Hz, 512 × 512 pixel image size.

GCaMP6f was imaged in *Pcp2-Cre* × Ai148 mice. In L*7-Tsc1* mutants with virally delivered Cre-dependent GCaMP6f (*n =* 2 mice), dendritic expression was sparse, and the few recorded dendrites showed calcium transients at low frequency (0.1–0.3 Hz) and prolonged half-decay times (around 1.72 s), indicative of significant Purkinje cell dysfunction, precluding reliable analysis of sensory-evoked activity.

##### Synchronization

Two forms of synchronization signal were sent from the behavior computer to the imaging computer during imaging. The first was a TCP/IP signal indicating animal and session identity. The second was an I2C-based signal routed through a National Instruments card (NI USB-8451). Signals were sent at multiple timepoints throughout each trial, delivering information corresponding to individual defined moments in the trial, which were then embedded in microscope image frames via ScanImage I2C functionality and retracted with imfinfo MATLAB function.

##### Imaging data processing

Imaging data were preprocessed, motion corrected and analyzed with Python package - CaImAn^64^ (https://github.com/flatironinstitute/CaImAn). Motion corrected movies were used for manual regions of interest (ROIs) selection. The manually selected dendritic ROI were subsequently refined using the Seed Constrained Nonnegative Matrix Factorization (CNMF) with external masks algorithm.^64^ After all ROI were refined with CNMF, the mean activity of all pixels in the ROI was computed for each frame, yielding raw time series data. ΔF/F0 was then computed from these raw traces, with baseline F0 being computed as the minimum of a median-filtered (1 s kernel) 12 s sliding window preceding each time point. Dendritic data analysis included trials of all cue-period durations.

##### Linear model with mixed effects (LME)

For LME analyses in Figure 3, we used the MATLAB function ‘fitlme’ to fit data to two linear mixed-effect models. We fitted the number of dendritic calcium transients Ccue during the cue periods in a single imaging session to an LME that incorporated the number of airpuffs in the trial P, the latent behavioral state S, and their interactions as fixed effects, with latent state 3 set as the reference. Mouse and dendrite labels were included as random effects, giving the following equation:

$${\text{C}}_{\text{cue}}=\beta_{\text{P}}\text{P}+\gamma_{\text{S}}+\gamma_{\text{C}}+\beta_{\text{P},\text{S}}\text{P}+\beta_{\text{P},\text{C}}\text{P}+\beta_{\text{P},\text{S},\text{C}}\text{P}+b_{\text{mouse}}+b_{\text{dendrite}}$$

Where the *β*s represent slope coefficients, the *γ*s represent constants, and *b*s represent random effects. For each imaging session, we also modeled the *Z*-scored number of dendritic calcium transients during the 800 millisecond post-decision period following the lick (*Z_decision_*), using as inputs the outcome type Err (categorical: Correct or Error trial), latent behavioral state S, dendrite cluster C, and their interactions as fixed effects. The reference conditions were correct outcome, latent state 3, and the non-clustered dendrites. The resulting fit equation was

$$Z_{\text{decision}}=\gamma_{\mathrm{Err}}+\gamma_{\text{S}}+\gamma_{\text{C}}+\gamma_{\mathrm{Err},\text{S}}+\gamma_{\mathrm{Err},\text{C}}+\gamma_{\mathrm{Err},\text{S},\text{C}}+b_{\text{mouse}}+b_{\text{dendrite}}$$

where these γ′s are different from those used in the C_cue_ model. *Z*-scores were calculated on a dendrite-by-dendrite basis by subtracting the mean overall response across all trials and dividing by the standard deviation. Model outputs are available on https://github.com/wanglabprinceton/accumulating-puffs-fastlearning.

##### *In vivo* electrophysiology

For acute recordings from awake behaving mice, animals were head-fixed over a freely rotating cylindrical treadmill and the craniotomy site was opened by removing the Kwik-Cast plug and then filled with saline. Recordings were performed using either silicon probes for neocortex or glass electrodes for cerebellum, as described below. Airpuffs to the whiskers were delivered by a pressure injector system (Toohey Spritzer, Toohey, Fairfield, NJ, USA) which received signals from a signal generator (Master-8; AMPI) with an intensity of 20 psi and a frequency of 1 Hz, except for experiments with continuous optogenetic activation throughout the entire cue and delay period, when airpuffs were delivered with a frequency of 0.2 Hz. Mice received unilateral airpuffs ipsilaterally to the recording site for Purkinje cells, anterior cingulate cortex, and anterolateral motor cortex, and contralaterally to the recording site for cerebellar nuclei and the barrel field of the somatosensory cortex. For recordings with optogenetic stimulation, light onset started at the same time as the airpuff for the duration of the airpuff (40 ms) unless indicated otherwise. In a subset of experiments (Figure S8) light started at the same time as the airpuff but remained on for longer (250 ms).

For neocortical recordings, a 64-channel silicon probe (Figures 5 and 6, S7, and S8; Neuronexus, A4x16-5mm-50-200-177 or A2x32-Poly5-10mm-20s-200-100) or a Neuropixels 1.0 probe (Figure 7) covered in Vybrant CM-DiI Cell-Labeling Solution (V22888; Invitrogen) was slowly placed above the craniotomy and lowered into the brain using a motorized micromanipulator (MP-225; Sutter Instrument Co.). The Neuronexus silicon probes were connected to two amplifier boards (RHD2132, Intan Technologies) using a dual headstage adapter (RHD2000, Intan Technologies). Recordings were made using an Open Ephys acquisition board at a sampling rate of 30 kHz. Neuropixels data were acquired using SpikeGLX (https://billkarsh.github.io/SpikeGLX/) and signals were digitized at 30 kHz. The probe was connected via a headstage to a PXIe data acquisition card (National Instruments, PXI-6133) in a PXI chassis (National Instruments, PXIe-1071), and connected to a computer via a PXIe interface (National Instruments, PXIe-8381). For all silicon probe recordings, high-pass filtering of the raw data at 300 Hz, common median referencing, and automatic spike sorting was achieved using Kilosort 2^66^ (https://github.com/cortex-lab/Kilosort). Spikes were further manually curated using the Phy GUI (https://github.com/kwikteam/phy).

Single-unit recordings of Purkinje neurons and cerebellar nuclei neurons were performed using borosilicate glass electrodes (1B100F-4, World Precision Instruments) with 1- to 2-μm tips, short for Purkinje cells or very long gradual tapers for cerebellar nuclei cells, and 3 to 12 MΩ impedance, fabricated on a pipette puller (P-2000, Sutter Instruments Co.) and filled with sterile saline. The electrode was lowered into the cerebellum using an electrode holder that was positioned at a 40 or 90° angle to the craniotomy and controlled by a motorized micromanipulator (MP-225; Sutter Instrument Co.). The obtained electrical signals were amplified with a CV-7B headstage and Multiclamp 700 B amplifier, digitized at 10kHz with a Digidata 1440 A and acquired in pClamp (Axon Instruments, Molecular Devices) in parallel with transistor-transistor logic (TTL) pulses from a signal generator (Master-8; AMPI) and with signal from pressure injector system (Toohey Spritzer, Toohey, Fairfield, NJ, USA).

Purkinje neurons were identified by the presence of complex spikes followed by a characteristic pause in simple spikes. For simple-spike analysis, we required a minimum recording duration of 10 s, and the presence of at least one complex spike to confirm the identity of the Purkinje cell. For complex-spike recordings the minimal recording duration was 100 s. In wild-type mice, recordings were more stable, resulting in equal numbers of recordings for simple-spike and complex-spike analysis. In mutant mice, recordings were less stable and reduced the relative number of analyzable complex-spike recordings. We selected airpuff-responsive recordings for further analysis. The cerebellar nuclei contain a high density of neurons that are deeper than, and well separated from, cerebellar cortical layers, and showed clear single unit spike activity. Spike detection was performed using custom code written in MATLAB 2019a. Latency was calculated as the time where the change in firing rate reached 30% of the first peak (minimal value within 100 ms after puff onset for simple spikes, maximum value within 100 ms after puff onset for all other areas). The area under the curve was calculated as the area between the baseline pre-stimulus firing rate and the firing rate of each unit in number of spikes per bin, for the time span indicated in the text.

#### Histology

Animals were anesthetized with an overdose of ketamine (400 mg/kg)/xylazine (50 mg/kg) (i.p.) and transcardially perfused using a peristaltic pump with phosphate buffered saline (PBS) with 10 mg/mL heparin (Sigma H3149-100KU), followed by chilled 10% formalin (Fisher Scientific). Brains were extracted from the skull after perfusion, postfixed overnight at 4°C, washed and stored in PBS at room temperature. To visualize the probe locations using the CM-DiI track, brains were cleared and imaged by the BRAIN CoGS histology core facility. All brains underwent the same abbreviated iDISCO+ clearing protocol as previously described.^35^ In short, after an overnight fix in 4% PFA, brains were rinsed in PBS at room temperature for four 30 min sessions. Immediately brains were dehydrated 1 h at each ascending concentration of methanol (20, 40, 60, 80, 100, 100%) and placed overnight in methanol at room temperature. The next day, they were being placed in 66% dichloromethane (DCM)/33% methanol for 3 h at room temperature. Brains were cleared with 100% DCM for two 15 min steps then placed in 100% benzyl ether (DBE). Brains were kept in fresh DBE prior to imaging and after for long-term storage. Tissue was imaged using a light-sheet microscope (Ultramicroscope II, LaVision Biotec., Bielefeld, Germany).

For quantification of Purkinje cells, Purkinje cells were stained with calbindin. Animals were transcardially perfused as described above, and after postfixation were stored in PBS at 4°C until sectioning. Whole brain sagittal sections were cut at 90 μm and collected in 0.1 M PBS. Sections were processed for immunohistology by washing with PBS and incubating for 1 h at room temperature in a blocking buffer (10% normal goat serum, 0.5% Triton in PBS) prior to a 2-day incubation at 4°C in PBS buffer containing 2% NGS, 0.4% Triton and the rabbit anti-calbindin-D-28 K primary antibody (C7354; Sigma-Aldrich St. Louis, MO, USA; 1:1000). Sections were subsequently washed in PBS, incubated for 2 h at room temperature in the PBS buffer with goat anti-rabbit Alexa Fluor 488-conjugated secondary antibody (A-11008; Thermo Fisher Scientific, MA, USA; 1:400), mounted on glass slides and covered with Vectashield. Images were acquired on the epifluorescent microscope Hamamatsu Nanozoomer. Using NDP.view2 Plus software, individual lobules were identified and Purkinje cells were assigned to lobules for counting.

#### Corticosterone measurements

Animals were food deprived for 12–24 h before blood collection. Immediately after receiving airpuffs to whiskers at 20–25 psi in a headfixed setup for 10–20 min, ~50 μL of blood was collected from the tail vein using a capillary tube, and then immediately disposed of in a heparin-coated 1.5 mL eppendorf tube. Samples were stored on wet ice for maximum 4 h, after they were centrifuged for 10 min at 4°C at 3000 rpm. Of each sample 2–10 μL of plasma was collected, placed in new non-coated 1.5 mL eppendorf tubes and stored at −80°C. For each animal, two duplicate samples of 1 μL each were used to determine plasma corticosterone levels using the Corticosterone ELISA Kit (K014; Arbor Assays, Ann Arbor, MI, USA) according to the manufacturer’s protocol. Plate reading was done using an Infinite 200Pro (Tecan Life Sciences, Morrisville, NC, USA) with i-control software. Results from both duplicates were averaged to get one final corticosterone measurement per animal.

#### Generalized linear model-hidden Markov model

The generalized linear model-hidden Markov model (GLM-HMM) is an analytical approach used here to identify underlying hidden behavioral states that underlie the progression of a long series of behavioral trials. The model combines a set of Bernoulli GLMs with a hidden Markov model.^25–27^ For each trial, an animal is modeled to have a latent state that governs its strategy to process information in order to make the binary choice of which side to lick. Each state corresponds to a specific GLM with a unique weight vector of input variables. At the beginning of a session, an initial state probability governs the likelihood of starting in a given state. Between trials, the transition matrix of HMM defines the probability to change from one state to another. The output of GLM-HMM in each trial is calculated as the probability of a Bernoulli response (i.e., the probability of a rightward lick) based on both the latent state of the current trial and the input variables. Delta cues (Δcues) is the number of airpuffs on the right side minus the number of airpuffs on the left side. Guide airpuffs (‘hints’) are included. Previous choice 1 is the animal’s choice on the previous trial. Previous choice 2 is the animal’s choice of the trial prior to the previous trial. Previous reward is the side of the reward on the previous trial. Bias is an offset constant in each state that represents the tendency to lick rightward independent of other input variables. The trials used to calculate the psychometric curve of a latent state are selected to have a posterior probability for that state larger than 0.8. The state occupancy of a certain state is calculated as the fraction of trials whose posterior state probabilities are greatest for that state. The model was trained with data from 22 wild-type mice used in a previous study^19^; such an approach for constructing a model using out-of-sample training data is commonly employed in order to avoid overfitting. As a validation of model generalization, we fit GLM-HMMs *de novo* to the behavioral data from each experimental group. These independently trained models converged to comparable maximal log likelihood values and recovered weight vectors and state structures that were highly similar to those obtained from the out-of-sample wild-type trained model. This confirms that the state definitions and derived parameters are robust to model initialization. Data were fitted using an expectation maximization algorithm with code adapted from https://github.com/Brody-Lab/venditto_glm-hmm.

#### Drift-diffusion modeling

The drift-diffusion model (DDM) framework is an analytical approach used to identify how mice accumulate multiple pulses of evidence from the whole cue period to make decisions. Here the previously used discrete evidence model was employed.^21^ Briefly, the DDM was developed based on Brunton et al.,^66^ where an accumulator *a*(*t*) tracks evidence during each trial. Right-side stimuli correspond to positive deflections, and left-side stimuli correspond to negative deflections. The trial’s choice is determined by the sign of the accumulator. The model incorporates noise (*σ*^2^_a_) in the accumulator, single-stimulus Gaussian variability (*σ*^2^*_s_*), and memory drift (*λ*). A negative λ results in leaky accumulation, while a positive λ causes instability. The model also includes bias and lapse parameters, with the latter accounting for random responses in a subset of trials. The model was fitted using PBupsModel Julia 0.6.3 package (https://github.com/misun6312/PBupsModel.jl). Parameter fitting utilized automatic differentiation to maximize model likelihood trial-by-trial and over time, with the Hessian matrix ensuring positive semidefiniteness for valid fits. Each fit used 1000 repetitions, randomly initializing parameters and omitting 20% of trials. Median parameters, standard deviation and 95% range were derived from these repetitions. Model accuracy was assessed using Bayesian Information Criterion through cross-validation, predicting held-out trial choices based on the best-fit parameters. To simulate the trial-wise trajectory of the accumulator for the latent states, best fit parameters of DDM (Table S2) were used to run the model on the same timing of the 5 left and 3 right discrete puffs with time steps of 15ms (Figure 3C).

#### Compositional data analysis

Traditional statistical methods that treat compositional data as independent and identically distributed can lead to misleading results because they ignore the inherent constraints imposed by compositional data; for instance, the fact that the sum of proportions always equals one. Compositional data analysis is a specialized framework for compositional data that directly addresses the proportional nature of the latent state data. Compositional analyses and ternary plots were conducted using the R package compositions^67^ (version 2.0–8). Zero or missing state occupancy values were imputed with the smallest positive normalized floating-point number. To ensure the total occupancy across the three states summed to one, this value was subtracted proportionally from the state with the largest occupancy. To graphically represent the dispersion structure of latent state data, centers were calculated using compositional means. Confidence regions were determined based on total variance and radius derived as 1.96 of the radial standard deviation on a log scale from a Fisher distribution with (D-1) and (N−D+1) degrees of freedom.

### QUANTIFICATION AND STATISTICAL ANALYSIS

#### Statistical analysis and presentation

Statistical parameters and tests used are indicated throughout the main text or in the figure legends. All further analysis was done with custom-written code in Python 3 using Spyder (https://www.spyder-ide.org/), and R version 4.3.3 (https://www.r-project.org/) using RStudio (https://www.rstudio.com/). For every figure, *p ≤ 0.05, **p ≤ 0.01, ***p ≤ 0.001. Boxes show median/interquartile range, and whiskers extend to the farthest data point that lies within 1.5 times the interquartile range from the end of the box. All figures show within-batch comparisons.

#### Effect size calculations

For comparison with previous literature, differences between *L7-Tsc1* mice and wild-type controls were calculated as the difference between means divided by the standard deviation. Standard deviation was taken as the standard error multiplied by the square root of the number of measurements. Measures from previous literature were vocalization, social preference, rotarod, and maze reversal,^18^ associative conditioning and gait,^17^ and open field.^29^

## Supplementary Material

1

2

3

Supplemental information can be found online at https://doi.org/10.1016/j.celrep.2026.117262.

## Figures and Tables

**Table:** KEY RESOURCES TABLE

REAGENT or RESOURCE	SOURCE	IDENTIFIER
Antibodies		
Rabbit anti-calbindin-D-28 K primary antibody	Sigma-Aldrich St. Louis, MO, USA	Cat# C7354; RRID: AB_476866
Goat anti-rabbit Alexa Fluor 488-conjugated secondary antibody	Thermo Fisher Scientific, MA, USA	Cat# A-11008; RRID:AB_143165
Critical commercial assays		
Corticosterone ELISA Kit	Arbor Assays	Cat# K014; RRID: AB_2877626
Experimental models: Organisms/strains		
Mouse: C57BL/6J	The Jackson Laboratory	JAX #000664; RRID:IMSR_JAX:000664
Mouse: *Pcp2-Cre*: B6.Cg-Tg(Pcp2-cre) 3555Jdhu/J	The Jackson Laboratory	JAX #010536; RRID:IMSR_JAX:010536
Mouse: Ai27D: B6.Cg-*Gt*(*ROSA*)*26Sor^tm27.1^* ^(CAG-COP4*H134R/tdTomato)*Hze*^/J	The Jackson Laboratory	JAX #012567; RRID:IMSR_JAX:012567
Mouse: Ai148: B6.Cg-*Igs7^tm148.1^*^(*tetO-GCaMP6f,CAG-tTA2*)*Hze*^/J	Hongkui Zeng, Allen Brain Institute	JAX #030328; RRID: IMSR_JAX:030328
Mouse: *Tsc1^flox/flox^*: *Tsc1^tm1Djk^*/J	The Jackson Laboratory	JAX #005680; RRID:IMSR_JAX:005680
Mouse: L7-Cre: B6.129-Tg(Pcp2-cre)2Mpin/J	The Jackson Laboratory	JAX #004146; RRID:IMSR_JAX:004146
Software and algorithms		
Code for accumulating puffs experiment	Deverett et al.^19^	https://github.com/wanglabprinceton/accumulating_puffs
Psychofit	Carandini and Wells^62^	https://github.com/cortex-lab/psychofit
FaceMap	Stringer et al.^63^	https://github.com/MouseLand/facemap
ScanImage 2015	Vidrio Technologies	RRID: SCR_014307
CaImAn	Giovannucci et al.^64^	https://github.com/flatironinstitute/CaImAn
SpikeGLX	N/A	https://billkarsh.github.io/SpikeGLX/
Kilosort 2	Pachitariu et al.^65^	https://github.com/cortex-lab/Kilosort
Phy	N/A	https://github.com/kwikteam/phy
Axon pCLAMP	Molecular Devices	https://www.moleculardevices.com/
MATLAB 2019	MathWorks	https://www.mathworks.com/products/matlab.html
NDP.view2 Plus	Hamamatsu	Cat# U12388-02; RRID: SCR_025177
i-control^™^ microplate reader software	Tecan Life Sciences	Cat#: STEM-LE-1445-LC; RRID: SCR_024562
GLM-HMM fitting code	N/A	https://github.com/Brody-Lab/venditto_glm-hmm
Discrete evidence drift-diffusion model	Deverett et al.^21^	N/A
PBupsModel	Brunton et al.^66^	https://github.com/misun6312/PBupsModel.jl
Julia 0.6.3	N/A	https://julialang.org/
Python 3	N/A	https://www.python.org/
R package compositions 2.0–8	van den Boogaart and Tolosana-Delgado^67^	http://www.stat.boogaart.de/compositions/
Spyder	N/A	https://www.spyder-ide.org/
R version 4.3.3	N/A	https://www.r-project.org/
RStudio	N/A	https://www.rstudio.org/
Other		
FAST-TRAC igloo and wheel, blue and amber	Bio-Serv	Cat# K3250/K3251/K3328/K3570/K3327
Mini yogurt drops	Bio-Serv	Cat# F7577
Isoflurane Inhalant Anesthetic	Med-Vet International	Cat# RXISO-250
Rimadyl (carprofen)	Zoetis	Cat# 141-199
Cannula, 316 S/S Hypo Tube 9 R GA. 0.1470/0.1490′ OD × 0.1150/0.1200′ ID × 0.0197′ long	Ziggy’s Tubes and Wires	Cat# 09R304-36
Norland Optical Adhesive 71	Norland Products	Cat# NOA71
Small round cover glass, #1 thickness, 3 mm	Warner Instruments	Cat# 64-0720
C&B Metabond^®^ Quick Adhesive Cement System	Parkell Inc.	Cat# S380
0.39 NA, Ø400 μm Core Multimode Optical Fiber	Thorlabs	Cat# FT400EMT
Ferrule ID Bore: 450μm; PFP LC 1.25mm OD Multimode 304 Stainless Ferrules	Precision fiber products	Cat# MM-FER2007-304-4500
High-Temp Epoxy 53 Formula Epoxy Bottle	Precision fiber products	Cat# PFP-353ND-8OZ
Vetbond Tissue Adhesive	3 M	Cat# 1469SB
Fiber Optic Cleaning Kit	Thorlabs	Cat# CKF
000-120 × 1/16 slotted Binding head machine screw 303 stainless steel	Antrin Miniature Specialties	Cat# AMS120/1 B
Kwik-Cast Low Toxicity Silicone Sealant	World Precision Instruments	Cat# KWIK-CAST
Master-8	A.M.P.I.	N/A
5mm 395-400nm UV Ultraviolet LED	EDGELEC	Cat# ED_YT05_U
High Torque Nema 17 Bipolar Stepper Motor 92oz.in/65Ncm 2.1 A Extruder Motor	Stepper Online	Cat# 17HS16-2004S-C5
PlayStation Eye Camera	Sony	Cat# MAIN-38675
Infrared 48LED illuminator	Serlium	Cat# Serlium2gmrkvdbax
Solenoid: standard two-way normally closed isolation valve	NResearch	Cat# 161T011
Miniature Air Pressure Regulator	ControlAir	Cat# 850
Speaker	Sony	Cat# XS-H20S
Custom two-photon microscope, movable objective microscope with resonant scanning	Sutter Instrument Company	N/A
Mai Tai Sapphire laser	Spectra-Physics	N/A
16× Nikon CFI LWD Plan fluorite objective, 0.80 NA, 3.0 mm WD	Nikon	Cat# N16XLWD-PF
Sonigel	Mettler Electronics Corp.	N/A
I2C/SPI Interface Device	National Instruments	Cat# USB-8451
Saline: 0.9% Sodium Chloride	Fisher Scientific	Cat# NC9054335
Toohey Spritzer Pressure System	Toohey Company	N/A
Silicon probe	NeuroNexus	A4x16-5mm-50-200-177
Silicon probe	NeuroNexus	A2x32-Poly5-10mm-20s-200-100
Neuropixels 1.0 probe	Neuropixels	N/A
Vybrant^™^ CM-DiI Cell-Labeling Solution	Invitrogen	Cat# V22888
Motorized Micromanipulator	Sutter Instrument	MP-225 A
Amplifier board	Intan Technologies	RHD2132
Dual headstage adapter	Intan Technologies	RHD2000
Acquisition board	Open Ephys	N/A
PXIe data acquisition card	National Instruments	PXI-6133
PXI chassis	National Instruments	PXIe-1071
PXIe interface	National Instruments	PXIe-8381
Borosilicate glass capillaries	World Precision Instruments	Cat# 1B100F-4
Micropipette puller	Sutter Instrument	P-2000
Headstage	Molecular Devices	CV-7B
Microelectrode Amplifier	Molecular Devices	Multiclamp 700 B
Low-noise Data Acquisition System	Molecular Devices	Digidata^®^ 1440 A
Ketamine	VetOne	Cat# 200-055
Xylazine	Akorn	Cat# 139-236
PBS pH 7.4	ThermoFisher	Cat# 10010072
Heparin	Sigma	Cat# H3149-100KU
Formalin	Fisher Scientific	Cat# 23-245685
Methanol	Caroline	Cat# 874195
Dichloromethane	Sigma	Cat# 270997-2 L
Benzyl alcohol	Sigma	Cat# 24122-1 L
Light-sheet microscope	LaVision Biotec	Ultramicroscope II
Normal Goat Serum	Sigma	Cat# G6767-100ML
Triton X-100	Sigma	Cat# T8787-50ML
VECTASHIELD^®^ Antifade Mounting Medium	Vector Laboratories	Cat# H-1000-10
Epifluorescent microscope	Hamamatsu	Nanozoomer
Plate reader	Tecan Life Sciences	Infinite 200 PRO

## Data Availability

All data that support the findings of this study will be made available upon reasonable request. Code used for data acquisition is available at https://github.com/wanglabprinceton/accumulating_puffs. Any additional information required to reanalyze the data reported in this paper is available from the lead contact upon request.

## Highlights

- Cerebellar manipulations in mice enhance sensory salience and accelerate learning
- Accelerated learning arises from prolonged occupancy in an on-task behavioral state
- Complex spikes simultaneously regulate neocortical activity, salience, and task state
- Cerebellum findings are consistent with a weak global coherence account of autism
