# Supplementary material for: Cerebellar acceleration of learning in an evidence-accumulation task
Source: Cell Rep. Author manuscript; Available in PMC 2026 May 24. (PMC13198533; doi:10.1016/j.celrep.2026.117262)
Supplement: 1 [file NIHMS2170802-supplement-1.pdf]

Cell Reports, Volume *45*

Supplemental information

Cerebellar acceleration of learning in an evidence-accumulation task

Marlies Oostland, Mikhail Kislin, Yuhang Chen, Tiffany Chen, Sarah Jo Venditto, Ben Deverett, and Samuel S.-H. Wang

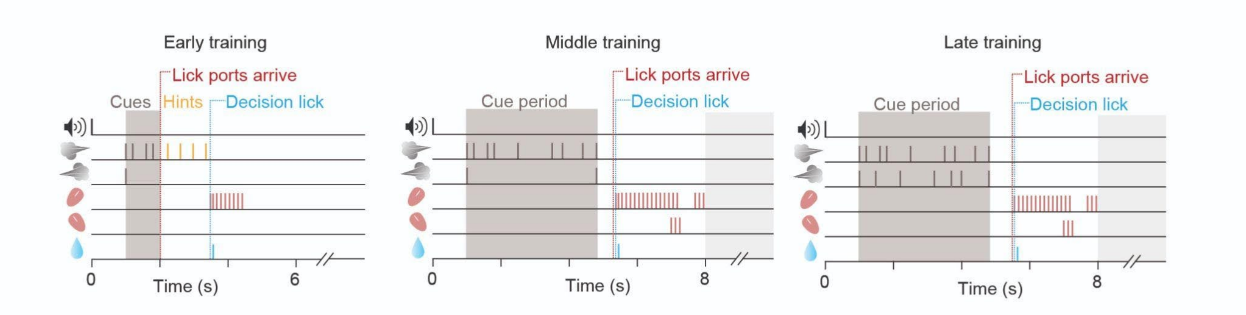

Figure S1, related to Figures 1 and 2. Three different training stages of the evidence-accumulation task

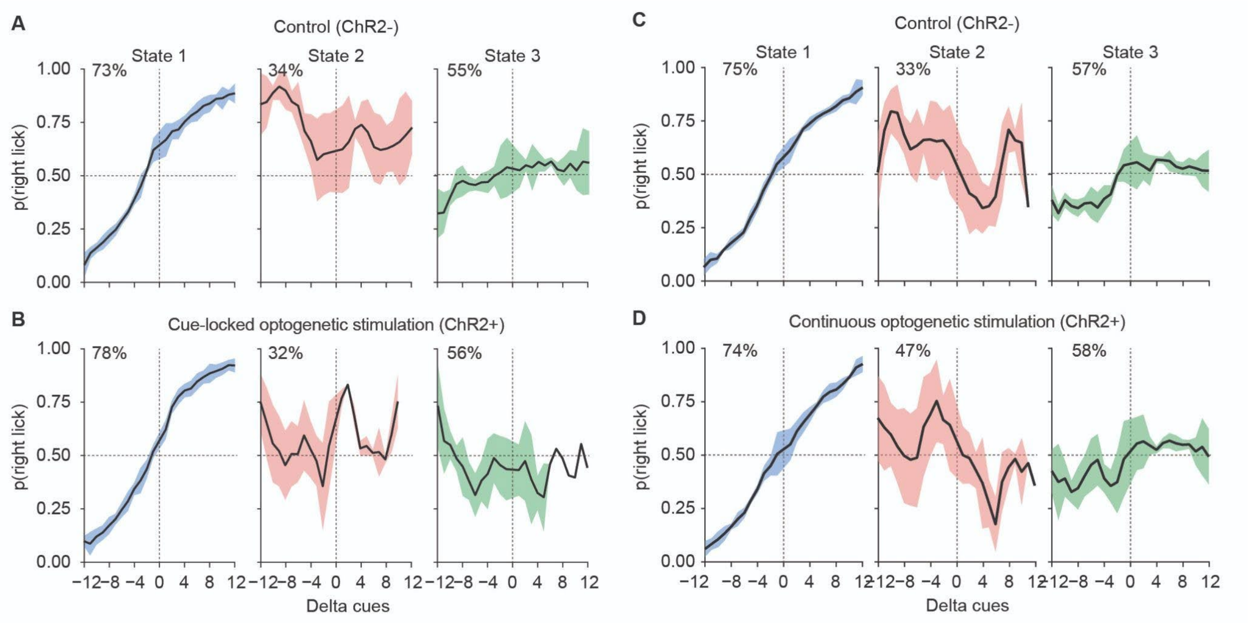

Figure S2, related to Figure 2. Psychometric curves in the three states remain the same for mice receiving cue-locked or continuous optogenetic stimulation

(A-D) Psychometric curves for the three states, averaged across all ChR+ mice receiving cue-locked optogenetic stimulation of Purkinje cells in crus I (B) and their ChR- littermates (A), ChR+ mice receiving continuous optogenetic stimulation of Purkinje cells in crus I (D) and their ChR- littermates (C). In the top left of each plot is the percentage correct over all trials in that state. Missing data points or data points without error bars indicate none or one animal at that data point due to low state occupancy. Shaded areas represent 1 s.d.

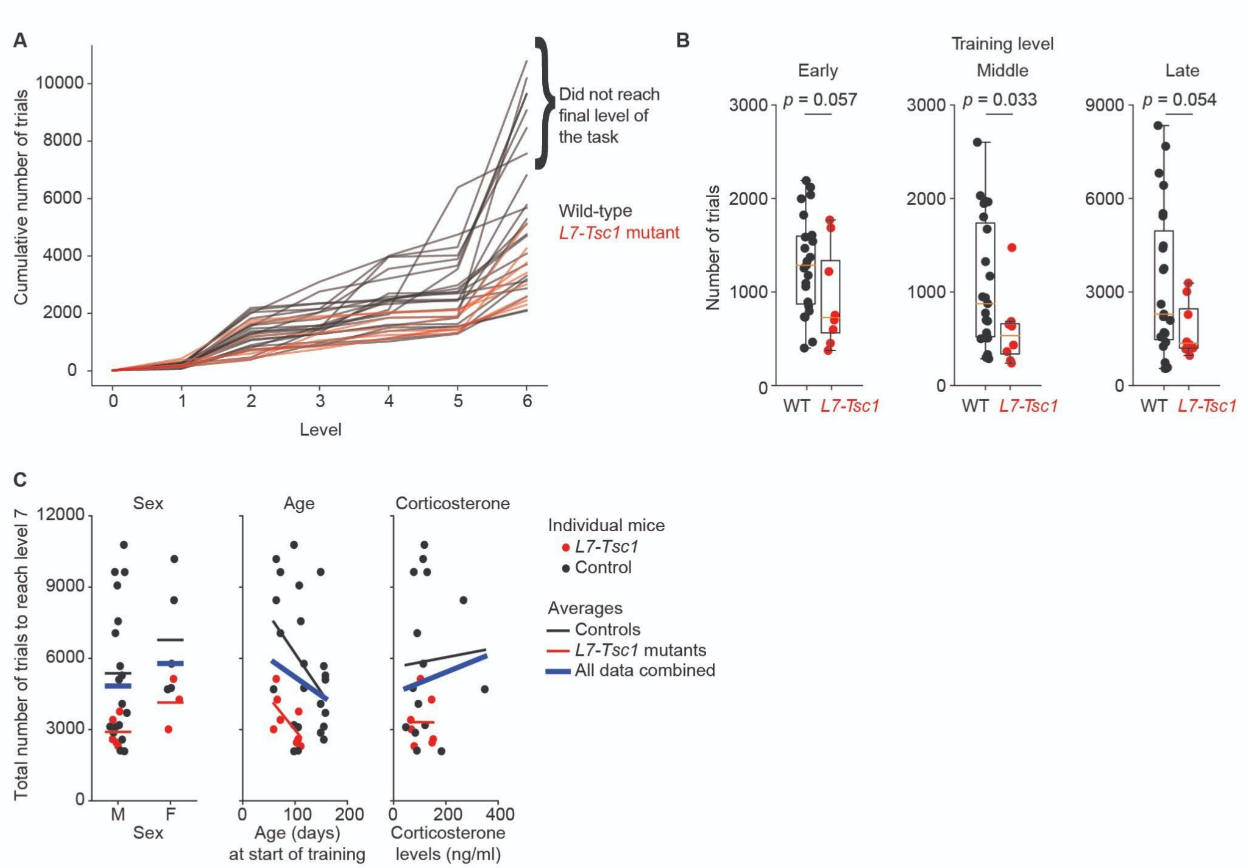

Figure S3, related to Figure 5. Learning trajectory for the evidence accumulation task for individual mice

(A) Cumulative number of trials needed to graduate to the next level for *L7-Tsc1* mutant mice (red) and control mice (black) in the evidence accumulation task. Each line indicates an individual animal. Six control animals did not reach the final level of the task. (B) Number of trials needed at early training levels (0, 1, and 2), middle (3 and 4) and late (5 and 6) levels for *L7-Tsc1* mutant mice (red) and control mice (black) in the evidence accumulation task. Learning was faster at middle stages of training (levels 3 and 4, $t_{(30)} = 1.9$, $p = 0.033$, one-tailed t-test; Figure S1B) and also showed a tendency to be faster at early (levels 0, 1, and 2; $t_{(30)} = 1.6$, $p = 0.057$) and late (levels 5 and 6, $t_{(30)} = 1.7$, $p = 0.054$) stages. (C) No correlation between the total number of trials to reach the final level of the evidence accumulation task and sex, age, or corticosterone levels. Numbers of animals are lower in the right panel than in the left and middle panels, as not all animals had blood samples taken to measure corticosterone levels.

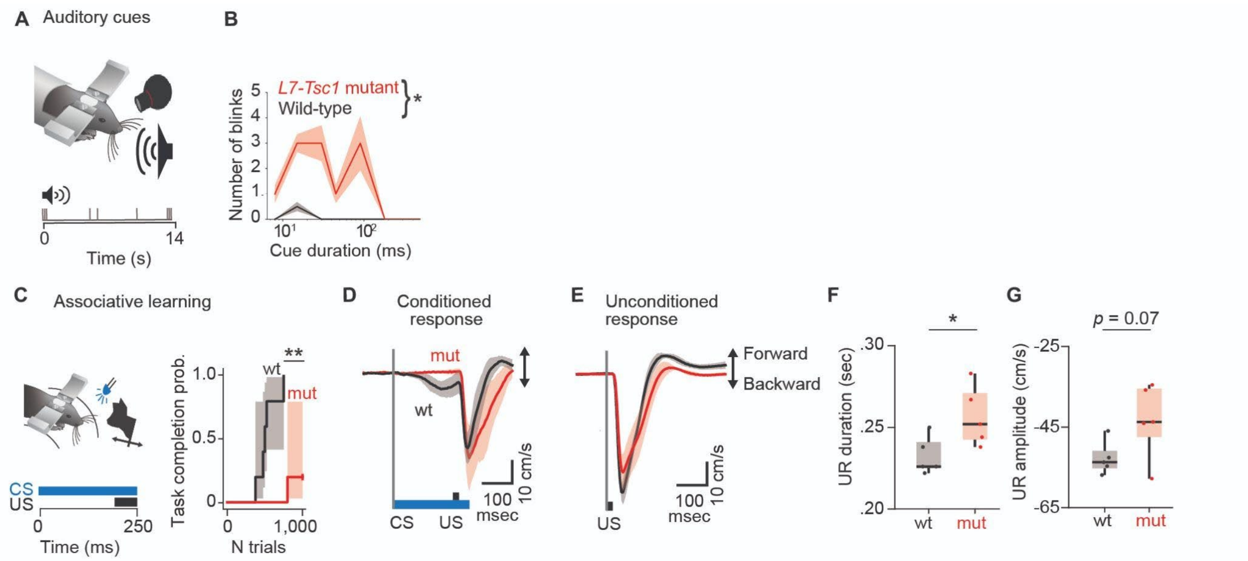

Figure S4, related to Figure 5. Impaired associative conditioning learning and increased sensory sensitivity in *L7-Tsc1* mice

(A) Schematic of sensory sensitivity tests with auditory cues. (B) Median number of eye blinks in response to auditory cues of different durations for *L7-Tsc1* mutant mice (n = 16) and wild-type littermates (n = 7). A two-way ANOVA indicates an effect of genotype ($F_{(1)}$ = 5.06, p = 0.026), but not of audio cue duration ($F_{(7)}$ = 1.697, p = 0.11) or an interaction effect ($F_{(7)}$ = 0.347, p = 0.93). (C) Impaired learning of the delayed tactile startle conditioning task (left) for *L7-Tsc1* mutant mice based on percentage backward CRs. Right: Kaplan-Meier estimator of probability of reaching the final level of task training for *L7-Tsc1* mutant mice. Shaded areas in Kaplan-Meier curves represent 95% confidence intervals. (D-E) Conditioned responses in session 5 (D) and unconditioned responses in sessions 1-5 (E) in the DTSC task for *L7-Tsc1* mutant animals (red) and control animals (black). Shaded areas indicate the estimated s.e.m. using median absolute deviation. (F) *L7-Tsc1* mutant mice have an increased duration of the unconditioned response (UR) in the DTSC task compared to wild-type controls ($F_{(1)}$ = 6.4, p = 0.04). (G) There is a trend toward larger amplitude of the unconditioned response (UR) in the DTSC task between *L7-Tsc1* mutant mice and wild-type controls ($F_{(1)}$ = 4.5, p = 0.07). * = $p \leq 0.05$, ** = $p \leq 0.01$.

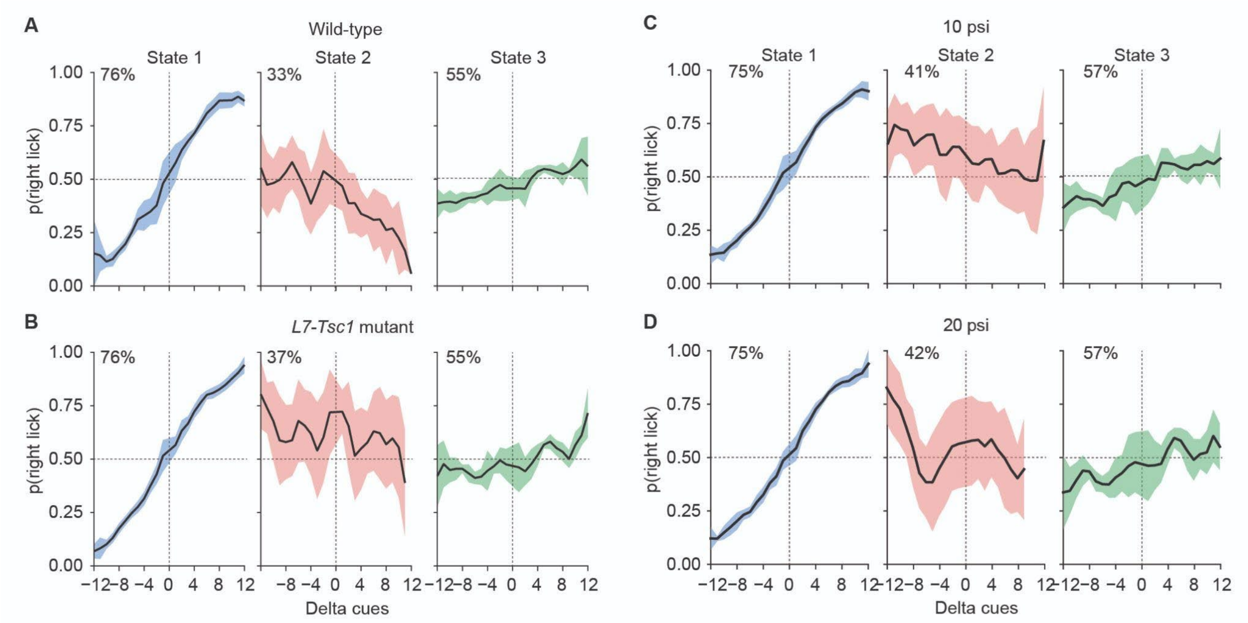

Figure S5, related to Figure 5. Psychometric curves in the three states remain the same for *L7-Tsc1* mice and for mice receiving stronger airpuffs to the whiskers

(A-D) Psychometric curves for the three states, averaged across all wild-type mice (A), *L7-Tsc1* mutant mice (B), mice receiving regular airpuffs of 10 psi (C) and mice receiving stronger airpuffs at 20 psi (D). In the top left of each plot is the percentage correct over all trials in that state. Missing data points or data points without error bars indicate none or one animal at that data point due to low state occupancy. Shaded areas represent 1 s.d.

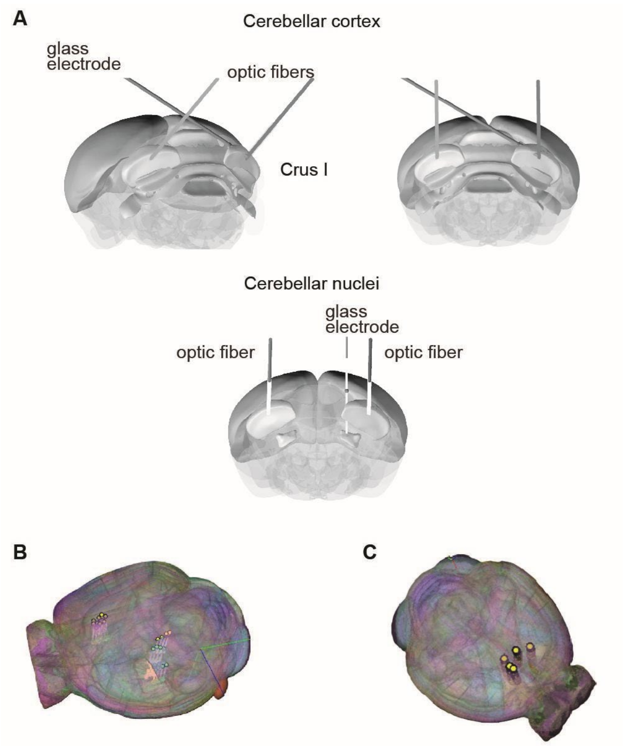

Figure S6, related to Figure 6. Recording locations in cerebellar cortex, cerebellar nuclei, and forebrain

(A) Orientation of the glass electrode during recordings in cerebellar cortex (top) and cerebellar nuclei (bottom) combined with bilateral optogenetic stimulation of crus I. (B) Examples of Neuronexus probe location in the forebrain for acute recordings in naive mice. (C) Visualization of the location of five Neuropixels probes targeted at anterior cingulate cortex for recording with simultaneous optogenetic stimulation of crus I while animals are performing the evidence accumulation task. B and C are made with Neuroglancer (https://github.com/google/neuroglancer).

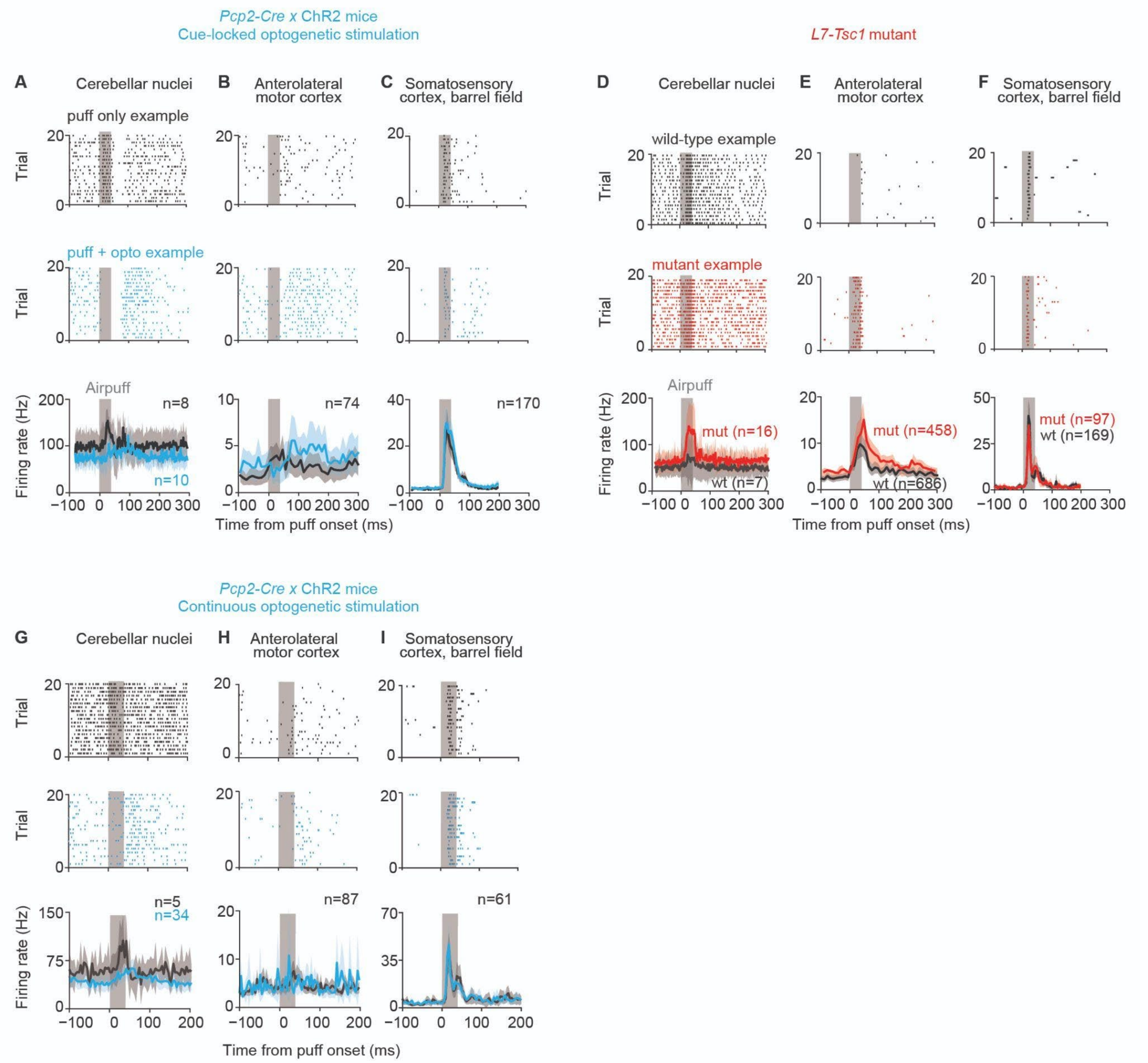

Figure S7, related to Figure 6. Neuronal responses to whisker puffs in anterolateral motor cortex and somatosensory cortex in naive mice with different (opto)genetic manipulations of the cerebellum.

(A) Example raster plots of cerebellar nuclei cells during 20 trials with only a whisker puff (top) or with a whisker puff paired with cue-locked optogenetic stimulation (middle), and average firing rates in response to an airpuff to the whiskers with or without cue-locked optogenetic stimulation of Purkinje cells. (B) Same as A, but for anterolateral motor cortex. (C) Same as A, but for the barrel field of the somatosensory cortex. (D) Example raster plots of cerebellar nuclei cells during 20 trials in a wild-type animal (top) or in a *L7-Tsc1* mutant animal (middle), and average firing rates in response to an airpuff to the whiskers in *L7-Tsc1* mutant animals and wild-type littermates. (E) Same as D, but for anterolateral motor cortex. (F) Same as D, but for the barrel field of the somatosensory cortex. (G) Example raster plots of cerebellar nuclei cells during 20 trials with only a whisker puff (top) or with a whisker puff paired with discounting

optogenetic stimulation (middle), and average firing rates in response to an airpuff to the whiskers with or without continuous optogenetic stimulation of Purkinje cells. (H) Same as G, but for anterolateral motor cortex. (I) Same as G, but for the barrel field of the somatosensory cortex.

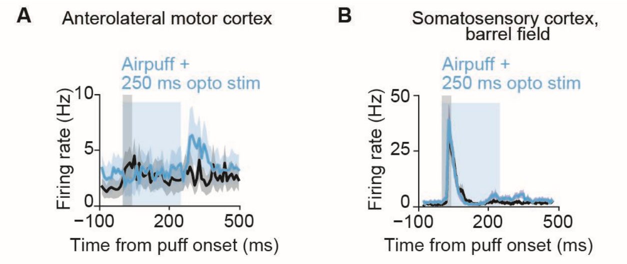

Figure S8, related to Figure 6. Increases in ALM firing, but not S1 firing, at the offset of the optogenetic stimulation of cerebellar crus I.

(A) Average firing rates in response to an airpuff to the whiskers with or without paired optogenetic stimulation for 250 ms of Purkinje cells in naive mice in anterolateral motor cortex. Optogenetic stimulation significantly increased the AUC 50 ms after the end of the 250 ms long optogenetic stimulation (airpuff only: mean AUC = 38±66, airpuff combined with optogenetic stimulation: mean AUC = 62±118, $t_{(73)} = -3.8$, $p = 0.0001$, paired t-test), but not 50 ms after the end of the 40 ms long airpuff (airpuff only: mean AUC = 36±75, airpuff combined with optogenetic stimulation: mean AUC = 50±135, $t_{(73)} = -1.7$, $p = 0.08$, paired t-test). (B) Average firing rates in response to an airpuff to the whiskers with or without paired optogenetic stimulation for 250 ms of Purkinje cells in naive mice in the barrel field of the somatosensory cortex. In S1, optogenetic stimulation also had an effect on the AUC 50 ms after the end of the 250 ms long optogenetic stimulation (airpuff only: mean AUC = 93±182, airpuff combined with optogenetic stimulation: mean AUC = 138±210, $t_{(169)} = -2.8$, $p = 0.006$, paired t-test), but not 50 ms after the end of the 40 ms long airpuff (airpuff only: mean AUC = 410±604, airpuff combined with optogenetic stimulation: mean AUC = 362±614, $t_{(169)} = 1.3$, $p = 0.21$, paired t-test). Shaded areas represent 95% confidence intervals.

Supplementary Table 1, related to Figures 1 and 2. Mice progress through eight different levels during learning of the evidence-accumulation decision-making task.

Level	0	1	2	3	4	5	6	7
**Audio cue, 1s before cue period onset**	Yes	Yes	Yes	Yes	Yes	Yes	Yes	Yes
**Bilateral puffs at start**	Yes	Yes	Yes	Yes	Yes	Yes	Yes	Yes
**Cue period duration (s)**	1	1	1	2.0, 2.8, or 3.8	3.8, or 1.5	3.8, or 1.5	3.8, or 1.5	3.8, or 1.5
**Contralateral distractor puffs**	No	No	No	No	No	No	Yes, 1:9	Yes, 1:4
**Bilateral puffs at end**	No	No	Yes	Yes	Yes	Yes	Yes	Yes
**Delay**	200 ms	200 ms	200 ms	200 ms	500 ms	800 ms	800 ms	800 ms
**Guide puffs (2.5 Hz) until animal licks**	No	Yes	Yes	No	No	No	No	No
**Need to lick on correct side for reward**	No (both sides rewarded)	Yes	Yes	Yes	Yes	Yes	Yes	Yes
**Does first lick need to be correct**	No	No	Yes	Yes	Yes	Yes	Yes	Yes
**Error trials punished with tone and timeout**	No	No	Yes	Yes	Yes	Yes	Yes	Yes
**Requirements to proceed to next level**	15 consecuti-ve rewards	at least 100 trials & 55% correct in window of 40 trials	at least 200 trials & 80% correct in window of 50 trials	at least 100 trials & 75% correct in window of 40 trials	at least 100 trials & 80% correct in window of 40 trials	at least 25 trials & 80% correct in window of 24 trials	at least 250 trials & 75% correct in window of 40 trials	<75% correct is acceptabl e

Supplementary Table 2, related to Figure 3. Best-fit drift diffusion model parameters for the three different latent behavioral states in C57BL/6 mice.

	Median	STD	SEM	95% range
**Accumulator noise ($puffs^2/s$)**				
**State 1**	0.01	0.69	0.14	0.00 - 2.15
**State 2**	26.36	33.86	10.21	2.42 - 89.96
**State 3**	10.16	36.32	7.4	0.23 - 97.71
**Sensory noise ($puffs^2/s$)**				
**State 1**	88.37	36.67	7.19	48.24 - 166.00
**State 2**	23.83	32.02	9.65	3.56 - 96.48
**State 3**	5.48	95.18	19.43	0.01 - 320.55
**Memory leak (s^{-1})**				
**State 1**	-0.17	0.17	0.03	-0.40 - 0.15
**State 2**	-1.20	2.93	0.88	-5.00 - 2.90
**State 3**	-0.95	1.65	0.34	-3.90 - 2.17
**Bias (Δ puffs)**				
**State 1**	-0.66	0.73	0.14	-2.36 - 0.67
**State 2**	0.52	3.71	1.12	-4.97 - 5.00
**State 3**	1.2	2.66	0.54	-5.00 - 5.00
**Lapse (probability)**				
**State 1**	0.00	0.04	0.01	0.00 - 0.11
**State 2**	0.65	0.39	0.12	0.00 - 1.00
**State 3**	0.00	0.19	0.04	0.00 - 0.52
